# Systematic characterization of Puerariae Flos metabolites *in vivo* and assessment of its protective mechanisms against alcoholic liver injury in a rat model

**DOI:** 10.3389/fphar.2022.915535

**Published:** 2022-08-30

**Authors:** Jialin Qu, Qiuyue Chen, Tianfu Wei, Ning Dou, Dong Shang, Dan Yuan

**Affiliations:** ^1^ Clinical Laboratory of Integrative Medicine, The First Affiliated Hospital of Dalian Medical University, Dalian, China; ^2^ Department of Traditional Chinese Medicine, Shenyang Pharmaceutical University, Shenyang, China

**Keywords:** Puerariae Flos, metabolic profile, alcoholic liver disease, bioinformatics, PPAR-α, MAO-A

## Abstract

Puerariae Flos, a representative homology plant of medicine and food for alcoholism, has a long history of clinical experience and remarkable curative effect in the treatment of alcoholic liver disease (ALD). However, its effective forms and hepatoprotective mechanisms remain unknown. In the present study, a strategy based on UPLC-QTOF MS combined with mass defect filtering technique was established for comprehensive mapping of the metabolic profile of PF in rat plasma, urine, bile, and feces after oral administration. Furthermore, the absorbed constituents into plasma and bile with a relatively high level were subjected to the network analysis, functional enrichment analysis, and molecular docking to clarify the potential mechanism. Finally, the therapeutic effect of PF on ALD and predicted mechanisms were further evaluated using a rat model of alcohol-induced liver injury and Western blot analysis. In total, 25 prototype components and 82 metabolites, including 93 flavonoids, 13 saponins, and one phenolic acid, were identified or tentatively characterized *in vivo*. In addition, glucuronidation, sulfation, methylation, hydroxylation, and reduction were observed as the major metabolic pathways of PF. The constructed compound–target–pathway network revealed that 11 absorbed constituents associated with the 16 relevant targets could be responsible for the protective activity of PF against ALD by regulating nine pathways attributable to glycolysis/gluconeogenesis, amino acid metabolism, and lipid regulation as well as inflammation and immune regulation. In addition, four active ingredients (6″-O-xylosyltectoridin, genistein-7-glucuronide-4′-sulfate, tectoridin-4′-sulfate, and 6″-O-xylosyltectoridin-4′-sulfate) as well as two target genes (MAO-A and PPAR-α) were screened and validated to play a crucial role with a good molecular docking score. The present results not only increase the understanding on the effective form and molecular mechanisms of PF-mediated protection against ALD but also promote better application of PF as a supplement food and herbal medicine for the treatment of ALD.

## Graphical Abstract

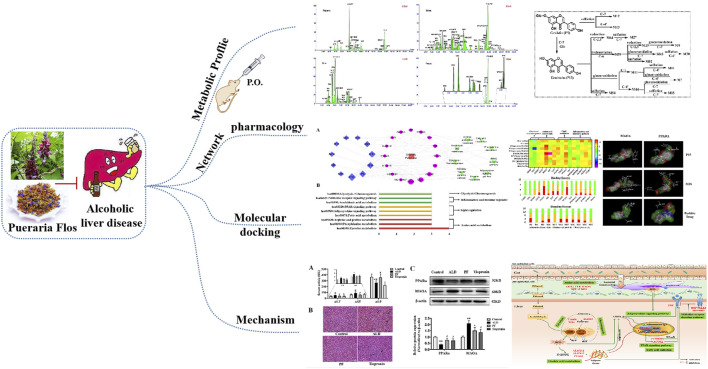

## Highlights

➢ Metabolic profile of Puerariae Flos (PF) extracts *in vivo* was firstly mapped.
➢ Phase II conjugated metabolites of isoflavonoids may be the effective forms responsible for the hepatoprotective effects of PF.
➢ PPAR-α and MAO-A involved in lipid regulation and amino acid metabolism were screened and validated to play a crucial role in the treatment of PF on alcoholic liver disease (ALD).
➢ A comprehensive mechanism for the multi-target and multi-pathway effects of PF on ALD was proposed.

## 1 Introduction

With the development of social economy, alcohol drinkers have been increasing in recent years. Alcohol abuse and alcoholism have emerged as common public health problems all round the world (Addolorato et al., 2016). As an important organ responsible for alcohol metabolism, the liver is the main target organ for alcohol toxicity. Accordingly, the incidence rate of alcoholic liver disease (ALD) caused by long-term heavy drinking is increasing year by year (Mathurin and Bataller, 2015). Therefore, exploring the effective treatment of ALD has become a particularly important issue which captures the attention of academic research. At present, the treatment of ALD largely relies on three medications or health products, namely, synthetic alcohol dehydrogenase inhibitor fomepizole (González-Santiago et al., 2009), commercial plant extracts of Camette *Silybum marianum* (L.) Gaertn. (Milk Thistle) and hydrodol tablets imported from Denmark and Australia (Rambaldi et al., 2005; Abenavoli et al., 2010), and an extract of oyster powder known as the Haiwang Jinzun tablet. However, their application is restricted due to side effects (hypotension, slow heartbeat, etc.), high price that depends on import, and safety problems incurred by the influence of fresh storage and marine pollution on raw materials. Overall, the drugs for treatment of ALD enjoy huge market capacity and potential in two respects. First, it has a stable and huge beneficiary population. Second, it is in a situation where imported drugs are dominant and domestic new drugs are obviously scarce.

There have been some records of “alcohol injury,” “alcohol jaundice,” and “alcoholism” in ancient Chinese medicine books for a long time. As an important part of traditional Chinese medicine, some ethnic and edible medicines have rich clinical experience and remarkable curative effect in the treatment of ALD, and they are valuable and prospective for drug research and development. Puerariae Flos (PF), known as “Ge-hua” in Chinese, is botanically from the dried flowers of *Pueraria montana* var. *thomsonii* (Benth.) M.R. Almeida or *P. montana* var. *lobata* (Willd.) Maesen and S.M. Almeida ex Sanjappa & Predeep. As a homology plant of medicine and food, it has been traditionally used to relieve toxic symptoms caused by excessive alcohol consumption, such as hangover, nausea, headache, and red face in China, Japan, and Korea for over 1,500 years (National Administration of Traditional Chinese Medicine "Chinese Materia Medica" Editorial Committee, 1999). Consequently, PF has been considered as the most representative antidote in traditional medicine. In clinical application, compound preparations such as Gehua Jiecheng Decoction (葛花解酲汤) and Jiusuyu (酒速愈) are its main forms, which have regulatory and protective effect on the nervous system and the liver caused by alcohol. Meanwhile, diet therapy is also the embodiment of the clinical application of PF. Products of medicine food homology including functional slimming food—PILLBOX Onaka, PF tea, and beverage have become increasingly popular among Asian people. Recent pharmacological studies showed that extracts and compounds from PF possessed various bioactivities, such as hepatoprotective (Xiong et al., 2010), hypoglycemic (Lee et al., 2000), hypolipidemic (Lee et al., 2000), estrogenic (Shin et al., 2006), and anti-inflammatory effects (Yuan et al., 2009). With respect to the chemical constituents of PF, more than 80 compounds have been isolated and identified to date (Qu, 2014), among which, isoflavanoids and triterpenoid saponins are two major types of constituents and play important roles in its pharmacological effects. In addition, five isoflavanoids kakkalide, irisolidone, 6″-O-xylosyltectoridin, tectoridin, and tectorigenin have been proved for potential medicinal values and regarded as important phytochemical markers for quality evaluation and differentiation between species under PF (Lu et al., 2013).

According to the concept of “effective forms” and “additive effect” of pharmacodynamics substances of TCMs, only the constituents and/or metabolites that are successfully assimilated into the circulatory system and maintain a considerable concentration level by additive effect in target organs may exert curative effects. In the past decade, the metabolism of PF isoflavanoids *in vitro* has been reported by some scholars (Hirayama et al., 2011). Our research group have also been devoting our efforts toward their ADME (absorption, distribution, metabolism, and elimination) characteristic by column separation, ultra-performance liquid chromatography/quadrupole time-of-flight mass spectrometry (UPLC-QTOF-MS), and NMR spectroscopy (Bai et al., 2010; Bai et al., 2011a; Bai et al., 2011b; Qu et al., 2012; Wang H. et al., 2013; Wang S. et al., 2013; Zhang et al., 2013; Qu et al., 2014; Shi et al., 2015; Zhang et al., 2015). A total of ten urinary and biliary metabolites have been isolated and structurally identified, and the plasma pharmacokinetics as well as urinary and biliary excretion of the conjugated metabolites was also determined in rats after oral administration. The results indicated that glucuronidation and/or sulfation after deglycosylation at the C-7 position was the major metabolic pathway of isoflavanoids from PF *in vivo*. In addition, kaikasaponin III, soyasaponin I, and kakkasaponin I were the most abundant saponins in PF and showed powerful protective effects against liver damage in the previous study, which are also responsible for the overall curative effects of PF (Kinjo et al., 1999). However, to the best of our knowledge, no reports have described the global metabolic profile of triterpenoid saponins or whole plant extract of PF *in vivo*. Moreover, pharmacological mechanisms and bioactive components of PF for the treatment of ALF are still not clear.

In this study, the absorbed and excreted prototypes and metabolites of the extract of *Pueraria montana* var. *thomsonii* (Benth.) M.R. Almeida in rat plasma, urine, bile, and feces were first characterized by UPLC-QTOF MS. Furthermore, with subsequent visualization of “ingredient–target–pathway–disease” association network constructed by using a network analysis and the binding interactions between key ingredients with targets performed by molecular docking simulation, the potential active components and underlying pharmacological mechanisms for the effect of PF on ALD were explored. Furthermore, the predicted key targets of PF against ALD were validated in an alcohol-induced liver injury rat model, which would promote better application of PF, which is a medical resource for developing a supplement food or an herbal medicine for the treatment of ALD (Graphic abstract).

## 2 Materials and methods

### 2.1 Chemicals, reagents, and materials

Puerariae Flos (Batch No. 161001) collected from Anhui Province was purchased from the Tong Ren Tang TCM store (Shenyang, Liaoning Province, China) in October 2017 and was authenticated as the flower of *Pueraria montana* var. *thomsonii* (Benth.) M.R. Almeida by Prof. Dan Yuan (Department of Traditional Chinese Medicine, Shenyang Pharmaceutical University). A voucher specimen was deposited at the authors’ laboratory. The reference compounds daidzein (16, Cat. No. NH010102) and luteolin (20, Cat. No. JOT-10088) were purchased from Funakoshi Co., Ltd. (Tokyo, Japan) and Chengdu Pufei De Biotech Co., Ltd. (Chengdu, China), respectively.

Moreover, 6″-O-xylosyltectoridin (10), tectoridin (12), genistein (23), tectorigenin (25), and irisolidone (32) were isolated from the extracts of the flowers of *Pueraria montana* var. *thomsonii* (Benth.) M.R. Almeida or *Pueraria lobate* (Willd.) Ohwi. in our previous studies (Yuan et al., 2009). The purities of these compounds evaluated using a HPLC photodiode array detector (PDA) were more than 95%.

HPLC-grade acetonitrile, methanol, and formic acid were supplied by Fisher Scientific Company Inc. (Fairlawn, NJ). Ultra-pure water (18.2 MΩ) was prepared using a Milli-Q water purification system (Millipore, Milford, MA, United States). All other reagents were of analytical grade and purchased from Shandong Yuwang Pharmaceutical Co., Ltd.(Yucheng, Shandong Province, China).

Rabbit monoclonal antibodies against peroxisome proliferator–activated receptor α (PPAR-α, Cat. No. ab126285), monoamine oxidase type A (MAO-A, Cat. No. A4105), and β-actin (Cat. No. AC026) were obtained from Abcam (Cambridge, MA, United States) and ABclonal Inc, (Wuhan, Hubei Province, China), respectively. A protein extraction kit was purchased from KeyGen Biotech Co., Ltd. (Nanjing, Jiangsu Province, China).

### 2.2 Preparation of Puerariae Flos extracts

Dried flowers of *Pueraria montana* var. *thomsonii* (Benth.) M.R. Almeida (1 kg) were weighed accurately and reflux-extracted twice with 80% EtOH (1:12 and then 1:10, w/v) for 1 h each time. After filtering with a six-layer absorbent gauze, the two filtered extracts were combined, concentrated under vacuum to 1 L (equal to 1 g crude herb/mL), and finally transformed into a freeze-dried powder.

Now, 50 mg of prepared powder was dissolved again with 10 ml of methanol/water (8:2, v/v) and extracted for 30 min under ultrasound. After centrifugation at 13,000 rpm for 10 min at 4°C and filtration through a 0.22-μm filter, 1.0 μL of filtrate was injected to UHPLC-QTOF MS for a qualitative analysis.

To calculate the administered dose, the contents of the three major ingredients were quantitatively determined by reported HPLC-UV using an external standard method (Zhang et al., 2009). The results indicated that the contents of 6″-O-xylosyltectoridin (10), tectoridin (12), and tectorigenin (25) in the extract were 112.5, 96.64, and 19.81 mg/g, respectively.

### 2.3 Sample collection and pretreatment *in vivo*

A total of nine male Sprague–Dawley rats (200 ± 20 g body weight and about 6- to 8-week-old) purchased from the animal center of Shenyang Pharmaceutical University were maintained in ambient houses (22 ± 2°C) with a 12-h light/12-h dark cycle. For acclimatization, rats were allowed soy-free food and water *ad libitum* in metabolic cages for 1 week before the experiments. The animals were divided into three groups at random: a dosed plasma collection group (*n = 3*), a dosed urine and feces collection group (*n = 3*), and a dosed bile collection group (*n = 3*). All animals were fasted for 12 h before the experiments and provided with free access to water and sugar over the period of sample collection. PF extracts were suspended in a 0.5% carboxy-methyl cellulose sodium salt aqueous solution with a concentration of 0.11 g/ml and administered by oral gavage at a dose of 1.1 g/kg body weight (equivalent to 200 mg tectoridin per kg) to rats. All experimental protocols were approved by the Ethics Review Committee for Animal Experimentation of Shenyang Pharmaceutical University (License number: SCXK (Liao) 2015-0001).

Serial blood samples (approximately 0.5 ml) were collected from the suborbital vein and placed in heparinized polythene tubes at 0, 0.5, 1, 1.5, 2, 3, 4, and 8 h after oral administration and then immediately centrifuged at 3,500 rpm for 10 min at 4°C to obtain plasma. Urine and bile samples were, respectively, collected at 0–2, 2–4, 4–8, 8–12, and 12–24 h after the dosing. Feces samples were collected at 0–12 and 12–24 h and then left in a cool and dry place to dry. The collected plasma, urine, and bile samples were mixed and pretreated using solid phase extraction, while the feces samples were extracted with ultrasound in methanol/water (75:25, v/v), according to our previous method (Zhang et al., 2013).

### 2.4 UPLC-QTOF MS analysis

The condition of chromatographic separation and mass detection was almost the same as those reported in the literature (Qu et al., 2014). Water containing 0.2% formic acid (solvent system A) and acetonitrile containing 0.2% formic acid (solvent system B) served as the mobile phase. The only difference is the change of elution gradient, which is listed as follows: 0–1 min, 5%–8% B; 1–8 min, 8%–13% B; 8–9.5 min, 13%–15% B; 9.5–11.5 min, 15% B; 11.5–14 min, 15%–16% B; 14–18 min, 16%–17% B; 18–30 min, 17%–65% B; 30–30.5 min, 65%–99% B; and 30.5–30.6 min, 99%–5% B.

### 2.5 Network analysis

#### 2.5.1 Identification of candidate targets of absorbed constituents and ALD

After identifying the absorbed and excreted ingredients of PF *in vivo* by UPLC-QTOF MS/MS, the chemical structure of absorbed constituents in plasma and bile with relative content higher than 3% was obtained as a SDF format by using ChemBioDraw Ultra 12.0 software, and then it was submitted to the Swiss Target Prediction platform (http://www.swisstargetprediction.ch/) to predict the most probable protein targets. The official gene names of top 100 targets with high matching degrees were selected for subsequent analysis. The targets associated with “alcoholic liver disease” were acquired from OMIM, TTD, CTD, GAD, DisGeNET, and GeneCards databases.

#### 2.5.2 The protein–protein interaction network analysis

The Search Tool for the Retrieval of Interacting Genes (STRING) database (https://string-db.org/) provides predicted PPI information as well as the data which have been experimentally confirmed. The version 11.0 of STRING was applied to acquire the PPI information, with the species limited to “*Homo sapiens*” and a confidence score > 0.9. These PPI targets were defined as core targets for further analysis.

#### 2.5.3 Enrichment analysis

The KEGG pathway analysis was performed by Database for Annotation, Visualization, and Integrated Discovery (DAVID, https://david.ncifcrf.gov/home.jsp, ver. 6.8) to find the signaling pathways related to candidate targets, and then the ALD-related pathways were selected.

#### 2.5.4 Network construction and analysis

The absorbed constituent–target–pathway–disease network was constructed by using the network visualization software Cytoscape 3.2.1, which supplies a method for data integration, analysis, and visualization for a complicated network analysis. In the network plot, a “node” signifies an ingredient, target, or pathway, and an “edge” represents the interaction among different targets. The “degree” of a node agreed with the number of its connected edges.

### 2.6 Molecular docking and dynamics

The Surflex docking program in Sybyl X2.0 was utilized to evaluate the binding energies and interactions between key active compounds and targets. The crystallographic structures of 16 target proteins were retrieved from the RCSB Protein Data Bank database (http://www.rcsb.org). The binding energy could be accomplished by the formation of binding pockets after preparation of ligands and receptors by removal of water molecules and original ligands, addition of hydrogen atoms, and repairmen of amino acids. “Total Score” was used as the indicator and positive correlation with docking preference. A score ≥ 4 was considered meaningful, which mean that there was a binding between the constituents and the targets.

### 2.7 Experimental validations of the pharmacological effects and the molecular mechanisms of PF against ALD

SD rats were randomly divided into four groups, including control group, alcoholic liver injury (ALD) model group, PF treatment (1.1 g⋅kg^−1^) group, and tiopronin treatment (60 mg⋅kg^−1^) group (*n* = 10 in each group). The medicine-treated rats were pretreated with PF extracts or tiopronin by intragastric administration twice daily for 1 week before the first dose of ethanol and at 1 h before each administration of ethanol doses for 4 weeks, whereas the rats in the control and model groups were given equivalent volume of 0.5% sodium carboxymethylcellulose. Except for the control group, the ALD model group was simultaneously induced by orally feeding 56% Erguotou wine (10 ml/kg/d) by gavage for 4 weeks.

Furthermore, 12 h after the final administration, blood was collected and centrifuged at 3,000 r/min for 15 min to obtain serum. Liver tissues were harvested and divided into two parts: one was fixed in 4% paraformaldehyde for histological observation and another was immediately stored in −80°C for the subsequent protein validation experiments.

The levels of alanine transaminase (ALT), aspartate transaminase (AST), and alkaline phosphatase (ALP) in the serum were measured using an automatic biochemistry analyzer (Hitachi, 7600-020, Tokyo, Japan). The middle lobe of liver tissues was collected, sectioned, and fixed in 4% paraformaldehyde for at least 24 h. After being dehydrated in ethanol and embedded in paraffin, a series of paraffin sections (5 μm) were stained with hematoxylin-eosin (H&E) for histopathological examination.

The liver tissues were lysed with a lysis buffer containing 1% PMSF, phosphatase inhibitors, and protease inhibitors and incubated in an ice bath for 30 min to extract total protein. The concentration of the extracted protein was measured by using the BCA quantitative method. Equal amount of protein was electrophoretically separated by 10% SDS-PAGE. After electrophoresis, the protein was transferred on PVDF membranes and then blocked in 5% milk with TBST for 2 h. The membranes were immersed in primary antibody of PPAR-α (1:1,000 dilution), MAO-A (1:1,000 dilution), and β-actin (1:50,000 dilution), respectively, at 4°C overnight; the next day, they were incubated with horseradish peroxidase–conjugated secondary antibody at room temperature for 2 h. Protein bands were detected with ECL Plus chemiluminescence reagent and quantified using ImageJ software (National Institutes of Health, USA). The values for each target protein were normalized to β-actin.

### 2.8 Statistical analysis

All values were expressed as mean ± standard deviation (SD). Differences between different groups were analyzed with one-way analysis of variance (ANOVA) using GraphPad Prism 8.0.1. The value of *p* < 0.05 was considered as statistically significant.

## 3 Results

### 3.1 Identification of PF metabolites *in vivo*

In order to identify the *in vivo* metabolites in rats, a total of 43 chemical constituents, including 22 isoflavonoids, 14 saponins, six flavonoids, and one phenolic acid were first identified or tentatively characterized in PF extracts by UPLC-QTOF MS based on our previous literatures (Lu et al., 2013) (Supplementary Figure S1, Supplementary Table S1). Consequently, the extracted ion chromatograms (EICs) were adopted to reduce the endogenous interferences from complex biological matrices and increase the sensitivity by using Metabolynx™ software combined with mass defect filtering (MDF) technique.

**FIGURE 1:**
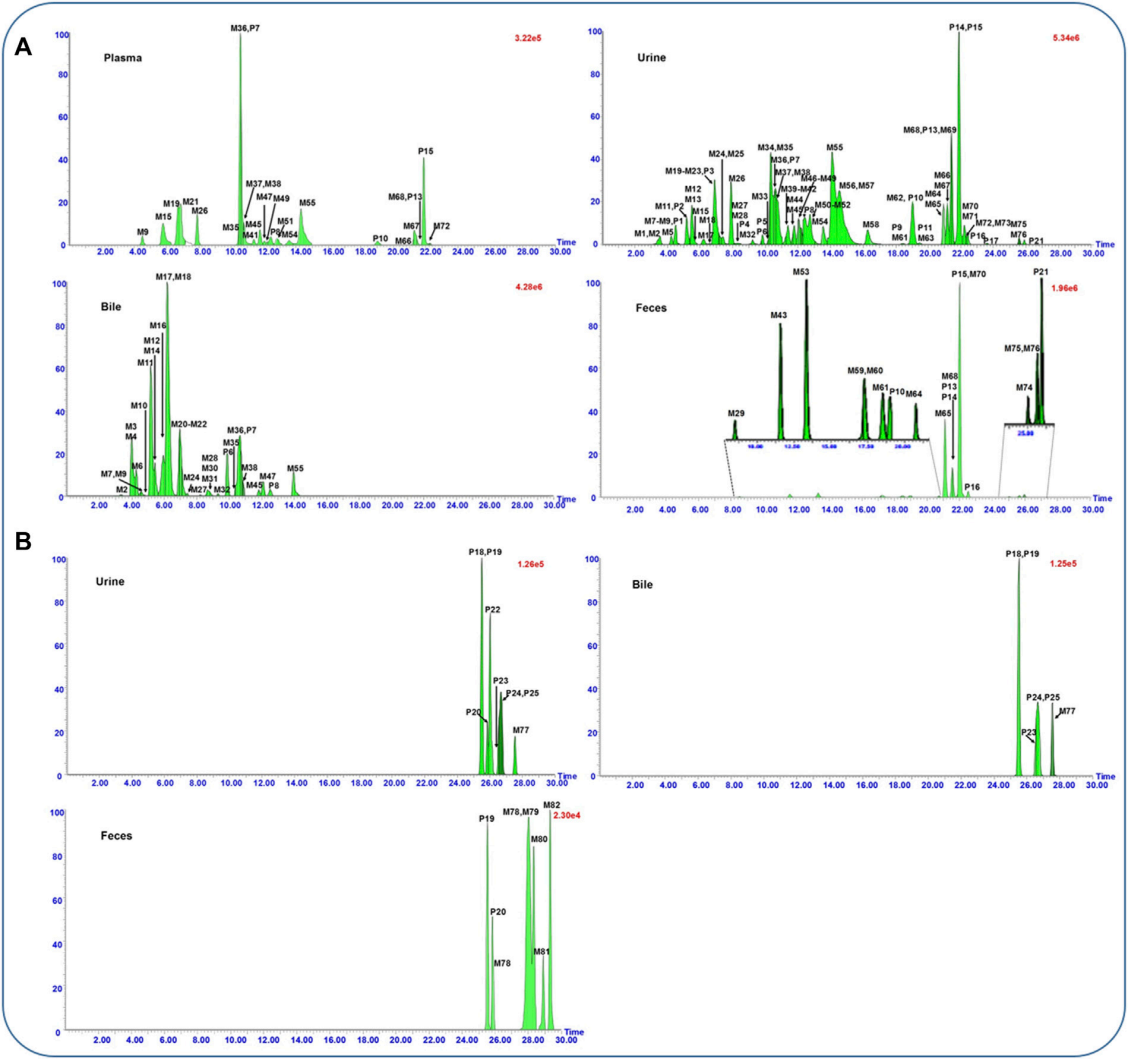
UPLC-QTOF/MS extracted ion chromatograms of flavonoid- **(A)** and saponin- **(B)** related metabolites in rat plasma, urine, bile, and feces samples after oral administration of the flower of *Pueraria montana* var. *thomsonii* (Benth.) M.R. Almeida extract.

In total, 25 prototype components and 82 metabolites, including 93 flavonoids, 13 saponins, and one phenolic acid, were obtained by comparing the extraction ion chromatograms of dosed rat biosamples (Figure 1) with control biosamples (Supplementary Figure S2). In addition, the peak area of each absorbed constituent was also recorded automatically using the MetaboLynx™ system. After summarizing the peak area of all constituents, the percentage calculated by the ratio of their peak area to the total peak area in each biological sample was described as relative content. The detail data of identified results are listed in Table 1. These compounds could be generally divided into two categories, namely, flavonoid- and saponin-related metabolites.

**TABLE 1:** Characterization of the metabolites in rat after oral administration of *P. montana* var. *thomsonii* (Benth.) M. R. Almeida extract by UHPLC-QTOF MS.

No.	t_R_	Formula	(M-H)^─^				Identification^a^	Relative content (%)^b^			
	(min)		Calculated	Observed	PPM	MS/MS fragments		P	U	B	F
M1	3.26	C_28_H_30_O_18_	653.1354	653.1315	−5.97	477,301,286	Dihydrotectorigenin-7G-4′G	—	0.03	—	—
M2	3.53	C_28_H_28_O_18_	651.1197	651.1194	−0.46	475,299,284	Tectorigenin-7G-4′G	—	0.50	0.16	—
M3	3.98	C_22_H_20_O_14_S	539.0496	539.0497	0.19	363,283,268	Glycitein-7G-4′-S	—	—	5.90	—
M4	4.1	C_28_H_30_O_17_	637.1405	637.1406	0.16	461,299,284	Tectoridin-7G	—	—	0.84	—
M5	4.2	C_22_H_22_O_13_	493.0982	493.097	−2.43	317,301,286	8-OH-Dihydrotectorigenin-7G	—	0.34	—	—
M6	4.26	C_22_H_22_O_13_S	525.0703	525.0704	0.19	445,283,268	Glycitin-4′S	—	—	2.92	—
M7	4.44	C_27_H_26_O_17_	621.1092	621.1105	2.09	445,269,133	Genistein-7G-4′G	—	0.17	0.18	—
M8	4.45	C_22_H_22_O_13_	493.0982	493.0974	−1.62	317,302	Dihydro-irilin D-7G	—	0.29	—	—
M9	4.51	C_21_H_20_O_12_	463.0877	463.0861	−3.46	287,259	6-OH-Dihydrogenistein-7G	1.18	0.44	0.28	—
P1	4.69	C_21_H_22_O_11_	449.1084	449.1083	−0.22	287,269	Dihydrokaempferol-7-O-glucoside	—	0.03	—	—
M10	4.76	C_28_H_30_O_17_	637.1405	637.1455	7.85	461,299,284	Tectoridin-4′G	—	—	0.06	—
M11	5.16	C_21_H_18_O_14_S	525.0339	525.0337	−0.38	349,269,133	Genistein-7G-4′S	—	1.27	15.57	—
P2	5.3	C_27_H_30_O_15_	593.151	593.1499	−1.85	285,151	Nicotiflorin	—	0.02	—	—
M12	5.38	C_21_H_20_O_13_S	511.0546	511.0534	−2.35	431,269,133	Genistin-7S	—	0.08	2.36	—
M13	5.48	C_16_H_14_O_10_S	397.0229	397.0228	−0.25	317,301,286	8-OH-Dihydrotectorigenin-7S	—	1.86	—	—
M14	5.53	C_22_H_22_O_14_S	541.0652	541.0621	−5.73	461,299,284	Tectoridin-7S	—	—	0.26	—
M15	5.79	C_21_H_18_O_14_S	525.0339	525.0361	4.19	349,269,133	Genistein-7S-4′G	6.05	0.02	—	—
M16	5.92	C_27_H_30_O_18_S	673.1075	673.1075	0.00	379,299,284	6″-O-Xylosyltectoridin-4′S	—	—	6.52	—
M17	6.21	C_22_H_22_O_14_S	541.0652	541.0643	−1.66	461,299,284	Tectoridin-4′S	—	0.34	31.08	—
M18	6.56	C_28_H_28_O_19_	667.1147	667.1157	1.50	491,315,300	Irilin D-7G-4′G	—	0.04	0.04	—
M19	6.71	C_22_H_20_O_15_S	555.0445	555.0441	−0.72	379,299,284	Tectorigenine-7G-4′S	12.17	22.71	—	—
M20	6.87	C_22_H_20_O_16_S	571.0394	571.0394	0.00	395,315,299,284	8-OH-Tectorigenine-7G-4′S	—	2.48	4.65	—
M21	6.89	C_22_H_20_O_11_	459.0927	459.0915	−2.61	283,268	Glycitein-7G	3.61	2.03	4.49	—
M22	7.14	C_22_H_22_O_15_S	557.0601	557.0587	−2.51	477,301,286	Dihydrotectorigenine-7G-4′S	—	0.35	0.83	—
M23	7.17	C_16_H_12_O_11_S_2_	442.9743	442.9748	1.13	363,283,268	Glycitein-7S-4′S	—	0.02	—	—
P3	7.28	C_22_H_22_O_10_	445.1135	445.1136	0.22	283,268,239	Glycitin	—	0.04	—	—
M24	7.37	C_22_H_20_O_11_	459.0927	459.0922	−1.09	283,268	Glycitein-4′G	—	0.11	0.15	—
M25	7.4	C_22_H_20_O_13_	491.0826	491.0817	−1.83	315,299,284	8-OH-Tectorigenine-7G	—	0.14	—	—
M26	7.88	C_16_H_14_O_6_	301.0712	301.0716	−0.66	286,257	Dihydrotectorigenin	4.40	2.94	—	—
M27	8.1	C_15_H_12_O_8_S	351.0175	351.0158	−4.84	271,253,225	Dihydrogenistein-7S	—	0.02	0.14	—
M28	8.36	C_16_H_12_O_12_S_2_	458.9692	458.9671	−4.58	379,299,284	Tectorigenin-7S-4′S	—	0.10	0.05	—
M29	8.5	C_15_H_12_O_6_	287.0556	287.0561	1.74	259	6-OH-Dihydrogenistein	—	—	—	0.13
M30	8.67	C_21_H_18_O_15_S	541.0288	541.0308	3.70	461,285,133	6-OH-Genistein-7G-4′S	—	—	0.80	—
M31	8.84	C_21_H_20_O_13_S	511.0546	511.0535	−2.15	431,269,133	Genistin-4′S	—	—	0.20	—
P4	9.02	C_27_H_30_O_16_	609.1456	609.1415	−6.73	301,285	Rutin	—	0.01	—	—
M32	9.21	C_22_H_20_O_16_S	571.0394	571.0386	−1.40	395,315,300	Irilin D-7G-4′S	—	0.24	0.21	—
P5	9.75	C_21_H_20_O_10_	431.0978	431.0961	−3.94	269,133	Genistin	—	0.02	—	—
P6 ^*c*^	9.82	C_27_H_30_O_15_	593.1506	593.1506	0.00	299,284,255	6″-O-Xylosyltectoridin	—	0.40	4.07	—
M33	10.15	C_22_H_20_O_11_	459.0927	459.0925	−0.44	283,268	Biochanin A-7G	—	0.16	—	—
M34	10.27	C_16_H_12_O_10_S	395.0073	395.0063	−2.53	315,299,284	8-OH-Tectorigenin-7S	—	0.19	—	—
M35	10.44	C_16_H_12_O_8_S	363.0175	363.0197	6.06	283,268	Glycitein-7S	0.31	4.26	0.84	—
M36	10.56	C_22_H_20_O_12_	475.0877	475.0863	−2.95	299,284	Tectorigenine-7G	31.64	3.25	8.23	—
P7 ^*c*^	10.62	C_22_H_22_O_11_	461.1084	461.1076	−1.73	299,284,255	Tectoridin	0.71	0.52	1.55	—
M37	10.74	C_16_H_12_O_8_S	363.0175	363.0174	−0.28	283,268	Glycitein-4′S	2.90	0.74	—	—
M38	10.8	C_22_H_20_O_12_	475.0877	475.0843	−7.16	299,284	Tectorigenine-4′G	1.27	2.26	1.00	—
M39	11.24	C_22_H_20_O_13_	491.0826	491.0819	−1.43	315,300	Irilin D-7G	—	0.16	—	—
M40	11.26	C_16_H_12_O_8_S	363.0175	363.0181	1.65	283,268	Biochanin A-7S	—	0.30	—	—
M41	11.39	C_21_H_18_O_11_	445.0771	445.0743	−6.29	269,133	Genistein-7G	0.80	0.80	—	—
M42	11.47	C_15_H_10_O_8_S	349.0018	348.9996	−6.30	269,151	6-OH-Daidzein-7S	—	0.05	—	—
M43	11.58	C_15_H_12_O_5_	271.0606	271.0599	−2.58	253,225	6-OH-Dihydrodaidzein	—	—	—	0.88
M44	11.68	C_21_H_18_O_11_	445.0771	445.0769	−0.45	269,133	Genistein-4′G	—	0.28	—	—
M45	11.76	C_21_H_18_O_12_	461.0720	461.0714	−1.30	285,133	6-OH-Genistein-7G	2.42	0.76	0.62	—
M46	12.02	C_16_H_14_O_10_S	397.0229	397.0219	−2.52	317,302	Dihydroirilin D-7S	—	0.27	—	—
M47	12.04	C_23_H_22_O_13_	505.0982	505.0963	−3.76	329,313,298,283,255	8-OH-Irisolidone-7G	0.55	1.11	1.53	—
M49	12.24	C_22_H_22_O_12_	477.1033	477.1028	−1.05	301,286	Dihydrotectorigenin-7G	—	0.16	—	—
**left**M48	12.24	C_16_H_14_O_10_S	397.0229	397.0212	−4.28	317,302	Dihydroirilin D-4′S	0.39	0.64	—	—
P8	12.43	C_21_H_20_O_11_	447.0927	447.0971	9.84	285,267	6-Hydroxygenistein-7-O-glucoside	1.56	0.28	0.84	—
M50	12.75	C_22_H_20_O_12_	475.0877	475.0872	−1.05	299,284,255	6-OH-Biochanin A-6G	—	1.25	—	—
M51	12.86	C_16_H_14_O_9_S	381.028	381.0269	−2.89	301,286	Dihydrotectorigenin-7S	1.51	2.50	—	—
M52	13.03	C_15_H_10_O_9_S	364.9967	364.9967	0.00	285,257	6-OH-Genistein-7S	—	0.19	—	—
M53	13.34	C_15_H_10_O_5_	269.045	269.0448	−0.74	151	6-OH-Daidzein	—	—	—	1.35
M54	14.08	C_15_H_10_O_8_S	349.0018	348.9989	−8.31	269,133	Genistein-7S	0.93	1.34	—	—
M55	14.2	C_16_H_12_O_9_S	379.0124	379.0116	−2.11	299,284	Tectorigenin-7S	12.10	8.75	2.64	—
M56	14.53	C_16_H_12_O_9_S	379.0124	379.0133	2.37	299,284	Tectorigenin-4′S	—	6.97	—	—
M57	15.01	C_16_H_12_O_10_S	395.0073	395.0068	−1.27	315,300	Irilin D-7S	—	1.00	—	—
M58	16.23	C_17_H_14_O_10_S	409.0229	409.0227	−0.49	329,313,298,283	8-OH-Irisolidone-7S	—	1.26	—	—
M59	17.19	C_15_H_10_O_6_	285.0399	285.0392	−2.46	257,229	6-OH-Genistein	—	—	—	0.26
M60	17.26	C_16_H_12_O_7_	315.0505	315.0511	1.90	299,284,255	8-OH-Tectorigenin	—	—	—	0.47
P9	18.44	C_16_H_12_O_7_	315.0505	315.0483	−6.98	300	Irilin D	—	0.06	—	—
M61	18.49	C_16_H_14_O_5_	285.0763	285.076	−1.05	270	Dihydroglycitein	—	0.03	—	0.54
P10	19.02	C_16_H_12_O_5_	283.0606	283.0614	2.83	268	Glycitein	0.68	2.62	—	0.45
M62	19.02	C_23_H_22_O_13_	505.0982	505.0937	−8.91	329,314	Iristectorigenin A-7G	—	0.27	—	—
P11 ^*c*^	19.47	C_15_H_10_O_6_	285.0399	285.0382	−5.96	133	Luteolin	—	0.09	—	—
M63	19.86	C_17_H_16_O_6_	315.0869	315.0845	−7.62	300,285,257	Dihydroirisolidone	—	0.01	—	—
P12	20.13	C_8_H_8_O_4_	167.0344	167.0345	0.60	108	Vanillic acid	0.51	0.04	—	—
M64	20.72	C_15_H_12_O_5_	271.0606	271.0589	−6.27	253,225	Dihydrogenistein	—	0.14	—	0.26
M65	20.92	C_16_H_14_O_6_	301.0712	301.0711	−0.66	286,257	6-OH-Dihydrobiochanin A	—	1.67	—	21.96
M66	21.14	C_17_H_14_O_10_S	409.0229	409.0215	−3.42	329,314	Iristectorigenin A-7S	0.43	0.54	—	—
M67	21.3	C_17_H_14_O_9_S	393.028	393.0269	−2.80	313,298,283,255	Irisolidone-7S	1.85	1.42	—	—
M68	21.41	C_16_H_12_O_6_	299.0556	299.0553	−1.00	284,255	Isotectorigenin	0.25	1.80	—	5.69
P13 ^*c*^	21.43	C_15_H_10_O_5_	269.045	269.0451	0.37	133	Genistein	0.59	3.02	—	2.74
M69	21.59	C_23_H_24_O_12_	491.119	491.1167	−4.68	315,300,285	Dihydroirisolidone-7G	—	0.03	—	—
P14	21.74	C_15_H_10_O_5_	269.045	269.0452	0.74	151	Apigenin	—	0.30	—	0.39
P15 ^*c*^	21.87	C_16_H_12_O_6_	299.0556	299.0548	−2.68	284,255	Tectorigenin	11.07	9.11	—	57.87
M70	22.13	C_17_H_14_O_7_	329.0661	329.0678	5.17	313,298,283,255	8-OH-Irisolidone	—	0.05	—	0.21
M71	22.2	C_23_H_22_O_12_	489.1033	489.1028	−1.02	313,298,283,255	Irisolidone-7G	—	0.68	—	—
M72	22.24	C_18_H_16_O_6_	327.0869	327.0883	4.28	313,298,283,255	4′,7-Di-methyltectorigenin	0.14	0.01	—	—
M73	22.3	C_16_H_12_O_6_	299.0556	299.0554	−0.67	284,255	6-OH-Biochanin A	—	0.35	—	—
P16	22.42	C_17_H_14_O_7_	329.0661	329.066	−0.30	314	Iristectorigenin A	—	0.24	—	1.55
P17	23.6	C_16_H_12_O_4_	267.0657	267.066	1.12	252	Formononetin	—	0.04	—	—
M74	25.03	C_16_H_14_O_5_	285.0763	285.0751	−4.21	270	Dihydrobiochanin A	—	—	—	0.13
P18	25.47	C_47_H_76_O_17_	911.5004	911.4987	−1.87	765,615,457,437	Astragaloside VIII	—	0.04	0.14	—
P19	25.52	C_48_H_78_O_18_	941.511	941.5088	−2.34	795,615,457,437	Soyasaponin I	—	0.15	0.36	0.76
M75	25.56	C_17_H_14_O_6_	313.0712	313.072	−0.66	298,283,255	Isoirisolidone	—	0.04	—	0.13
M76	25.69	C_16_H_12_O_5_	283.0606	283.0605	−0.35	268	Biochanin A	—	0.21	—	0.25
P20	25.86	C_47_H_76_O_17_	911.5004	911.5	−0.44	765,615,457,437	Soyasaponin II	—	0.04	—	0.29
P21 ^*c*^	25.89	C_17_H_14_O_6_	313.0712	313.0714	0.64	298,283,255	Irisolidone	—	0.16	—	0.64
P22	26.01	C_48_H_78_O_17_	925.5161	925.517	0.97	779,599,441,439	Kaikasaponin III	—	0.16	—	—
P23	26.57	C_42_H_68_O_13_	779.4582	779.4576	−0.77	617,439	Kaikasaponin I	—	0.05	0.13	—
P24	26.66	C_47_H_76_O_16_	895.5055	895.5039	−1.79	599,441,439	Kakkasapnin I	—	0.05	0.11	—
P25	26.75	C_47_H_76_O_16_	895.5055	895.5021	−3.80	599,441,439	Baptisiasaponin I	—	0.05	0.10	—
M77	27.54	C_41_H_66_O_12_	749.4476	749.4461	−2.00	587,411,409	Demethyl-22-dehydroxyl- kaikasaponin I	—	0.03	0.16	—
M78	27.92	C_36_H_60_O_9_	635.4159	635.4177	2.83	459,438	Reduct-soyasapogenol B-3-β-D-glucuronide	—	—	—	0.14
M79	28.03	C_31_H_50_O_3_	469.3682	469.3668	−2.98	455,439,437	Methyl-soyasapogenol E	—	—	—	1.48
M80	28.32	C_30_H_48_O_5_	487.3423	487.3398	−5.13	471,455,439	1, 21-Dihydroxyl-soyasapogenol E	—	—	—	0.55
M81	28.89	C_37_H_62_O_9_	649.4316	649.4307	−1.39	473,459,441,439	Methyl-reduct-soyasapogenol B-3-β-D-glucuronide	—	—	—	0.23
M82	29.31	C_30_H_50_O_4_	473.3631	473.3635	0.85	457,437	1-Hydroxyl-soyasapogenol B	—	—	—	0.65

a G, glucuronide; S, sulfate; and OH, hydroxylation.

b P, plasma samples; U, urine samples; B, bile samples; and F, fecal samples.

c Components identified with reference compounds comparison.

#### 3.1.1 Metabolites associated with flavonoids

A total of 93 flavonoids and their metabolites were screened out from the dosed samples, with 16 of them elucidated as prototypes and others assigned as metabolites. Among the prototypes, eight components that were almost consistent with our previous study belonged to aglycones (P9∼P11, P13∼P17, and P21) and glycosides (P1∼P8), respectively. In addition, five (P7∼P8, P10, P13, and P15), 25 (P1∼P25), three (P6∼P8), and six (P10, P13∼P16, and P21) prototype components were observed separately in rat plasma, urine, bile, and feces samples. As for the metabolites, glucuronidation, sulfation, methylation, hydroxylation, and reduction were their major metabolic pathways. 76 metabolites, including 24 sulfates, 21 glucuronides, 17 aglycones, eight glucuronide–sulfates, four diglucuronides, and two disulfates were identified (Figure 1A).

**FIGURE 2:**
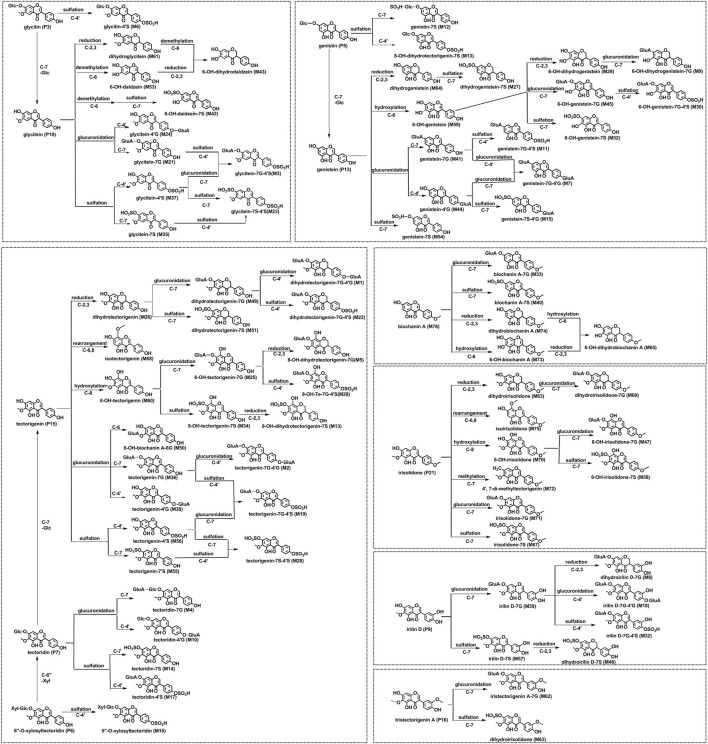
Proposed metabolic pathways of flavonoid-related metabolites in rats after oral administration of the flower of *Pueraria montana* var. *thomsonii* (Benth.) M. R. Almeida extract.

As for the aglycone skeleton, MS^2^ spectra with high energy showed characteristic ^1,3^ A^−^ and ^1,3^ B^−^ ions origin from a retro-Diels-Alder (RDA) cleavage of the C ring as well as losses of CH_3_ (15 Da), O (16 Da), H_2_O (18 Da), CO (28 Da), CO_2_ (44 Da), and/or combination of the fragments mentioned before. Meanwhile, the reduction at 2,3-double bond of the C ring and rearrangement between C-6 and C-8 positions were also common in the *in vivo* metabolic pathway of PF flavonoids according to the related literature (Bai et al., 2010; Bai et al., 2011a). Correspondingly, the metabolic pathway of flavonoids from PF *in vivo* was outlined, as shown in Figure 2. The results indicated that irilin D (P9), glycitein (P10), genistein (P13), tectorigenin (P15), iristectorigenin A (P16), and irisolidone (P21), which were the aglycone of flavonoids constituent in PF extracts, were the key prototype components in the metabolic process.

#### 3.1.2 Metabolites associated with saponins

Triterpenoid saponins found in PF are another major group of bioactive components. In addition, 13 saponins, sharing a basic chemical skeleton but with different moieties at C-3, C-22, and C-28, were identified and tentatively characterized based on their mass spectra and literature reports (Gao et al., 2007; Liang et al., 2014; Yu et al., 2016) (Figure 1B).

Compounds P18∼P20, M78, and M81∼M82 could be grouped because they possess the same aglycone soyasapogenol B (C_30_H_50_O_3_) as the aglycone, and they presented similar fragmentation pathways. P18 and P20 with precursor ions detected at *m/z* 911 (M−H)^−^ are a pair of isomeric with an identical elemental composition of C_47_H_76_O_17_. Both displayed a series of characteristic fragment ions at *m/z* 765, 615, 457, and 437 by simultaneously losing sugar units including 146 Da (rhamonose), 132 Da (arabinose, xylose), 176 Da (glucuronosyl), and 18 Da (H_2_O) at the site of C-3. They were ascribed to astragaloside VIII and soyasaponin II, respectively (Lu et al., 2013). Similarly, P19 was identified as soyasaponin I according to our previous reports (Lu et al., 2013). The metabolites M78 and M81 exhibited the protonated molecular ion at *m/z* 635.4177 (C_36_H_60_O_9_) and *m/z* 649.4307 (C_37_H_62_O_9_), which were 2Da (2H) and 16 Da (CH_2_+2H) higher than that of the soyasapogenol B-3-β-D-glucuronide, suggesting that both were reduced and methylated metabolites. The reduction at 12,13-double bond of the C ring is a metabolic pathway for saponins such as α-hederin (Liang et al., 2014), which was also reduced to hydrogenated metabolite in rat feces by the gut microflora. Accordingly, M78 and M81 were tentatively identified as reduct-soyasapogenol B-3-β-D-glucuronide and methyl-reduct-soyasapogenol B-3-β-D-glucuronide based on the aforementioned researches. Similarly, M82 with *m/z* 473.3635 (C_30_H_50_O_4_) was 16 Da (O) higher than that of aglycone, which was identified as 1-hydroxyl-soyasapogenol B because C-1 was the active site according to the related research (Yu et al., 2016).

P22∼P25 and M77 could be grouped because they possess the same aglycone sophoradiol. P22∼P25 were identified as kaikasaponin III, kaikasaponin I, kakkasaponin I, and baptisiasaponin I by comparison with the identified constituent in PF extracts, respectively (Lu et al., 2013). M77 exhibited a protonated molecular ion at m/z 749.4461 (C_41_H_66_O_12_), which was 30 Da (-CH_2_-O) lower than that of kakkasaponin I (P23). Thus, it was demethylated and dehydroxylated metabolite of P23. Since there is only one hydroxyl substituted at C-22, M77 was deduced as dimethyl-22-dehydroxyl-kakkasaponin I. Analogously, M79 and M80 were identified as methylated and C1, C21-dihydroxyl of aglycone soyasapogenol E based on the similar metabolic pathway of saikosaponin G and glycyrrhetinic acid (Gao et al., 2007; Yu et al., 2016).

### 3.3 Compound–target–pathway network construction

**FIGURE 3:**
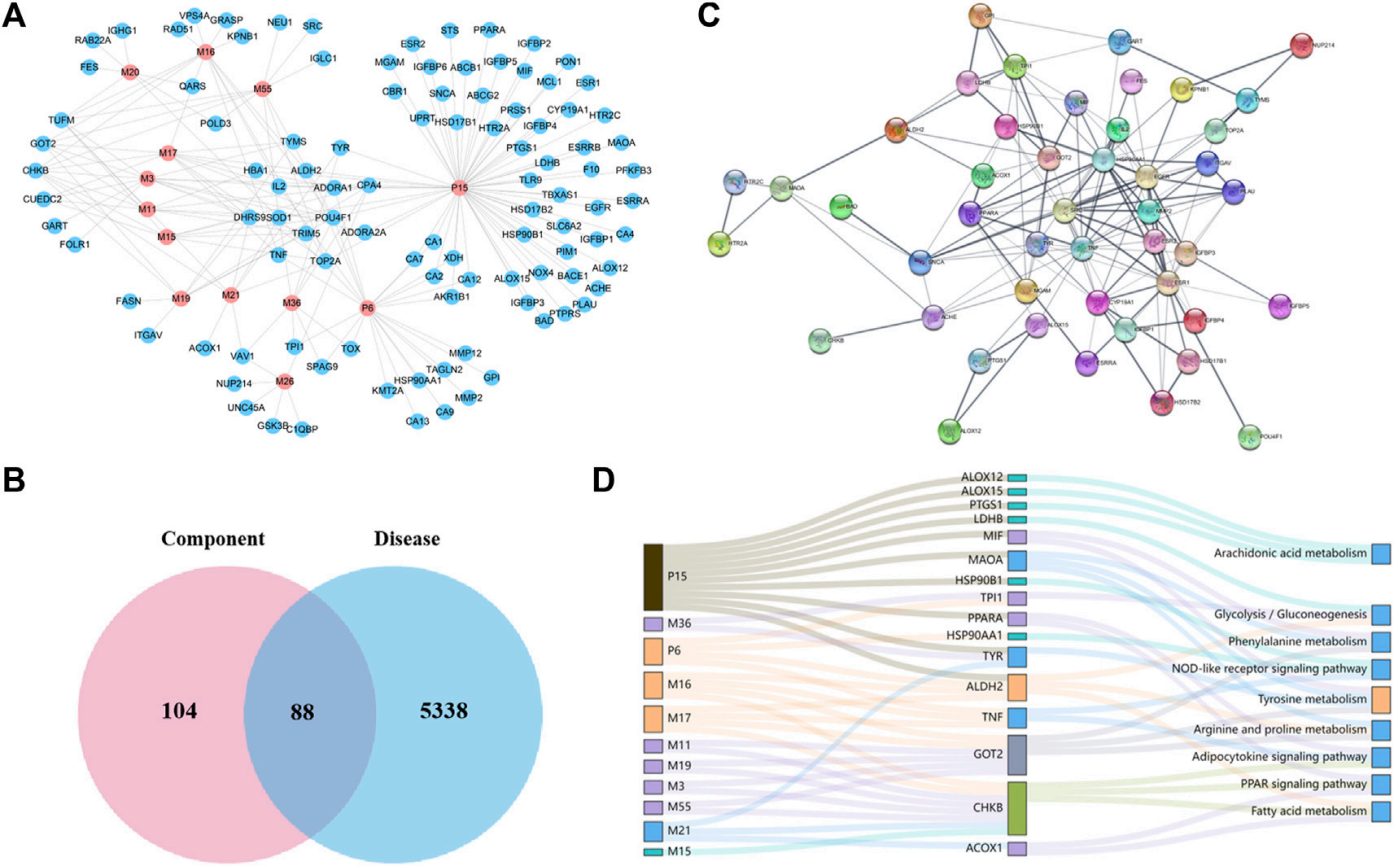
Network construction and correlation analysis. **(A)** Distribution characteristic of 13 candidate-absorbed components and their predicted targets; **(B)** Venn analysis of targets between 13 candidate components and ALD; **(C)** PPI network of 47 merged targets according to the STRING database; **(D)** interaction network of absorbed constituents, candidate targets, and enriched pathways. A node stands for a constituent (left), a target (middle), or a pathway (right); the interactions of two nodes were represented by a line, and the bigger size of a node, the higher the degree.

In order to understand important effective components, the relative content of each metabolite was calculated by area normalization and expressed as the percentage of its peak area to the total peak areas in each kind of biosamples (Table 1). A total of 13 candidate components that detected in rat plasma and/or bile samples with relative content more than 3% were screened for the further network analysis. In all, 104 protein targets associated with the 13 constituents were retrieved from the Swiss Target Prediction platform after eliminating the overlaps, and a component–target network was constructed (Figure 3A). Their detail information is shown in Supplementary Table S2. Similarly, 5338 ALD-related targets obtained from OMIM, TTD, CTD, GAD, DisGeNET, and GeneCards databases were collected after searching, integrating, and de-duplicating steps (Supplementary Table S3).

To acquire the candidate targets of PF against ALD, aforementioned constituent targets and disease targets were intersected, and 88 intersection targets were obtained (Figure 3B). Then the protein–protein interaction (PPI) analysis aiming at more crucial targets was carried out based on the aforementioned 88 targets by using the STRING database. Accordingly, 47 candidate targets with confidence scores greater than 0.9 were screened out (Figure 3C) and subjected to the KEGG pathway enrichment to elucidate their molecular mechanisms. As a result, nine signaling pathways (excluded cancer pathways) were involved and enriched from 16 of 47 targets (Figure 3D), which could be sorted into four groups according to their biological functions: (1) glycolysis/gluconeogenesis-related targets (LDHB, TPI1, and ALDH2); (2) amino acid metabolism–related targets (ALDH2, GOT2, MAOA, MIF, and TYR); (3) lipid regulation–related targets (ALDH2, ACOX1, CHKB, PPARA, and TNF); and (4) inflammation and immune regulation–related targets (TNF, HSP90AA1, HSP90B1, ALOX12, ALOX15, and PTGS1). Furthermore, we mapped the 16 targets into components and obtained 11 absorbed components (P6, P15, M3, M11, M15∼M17, M19, M21, M36, and M55) (Figure 3D).

### 3.4 Molecular docking

A docking analysis was performed to evaluate the relationship between the active components and potential targets that were predicted by the network analysis. The aforementioned absorbed 11 ingredients were selected as candidate components to dock with the 16 relevant targets that were screened by the network analysis. Consequently, a heat map performed by GraphPad 8.0 software was present for intuitively describing the receptor–ligand interactions.

**FIGURE 4:**
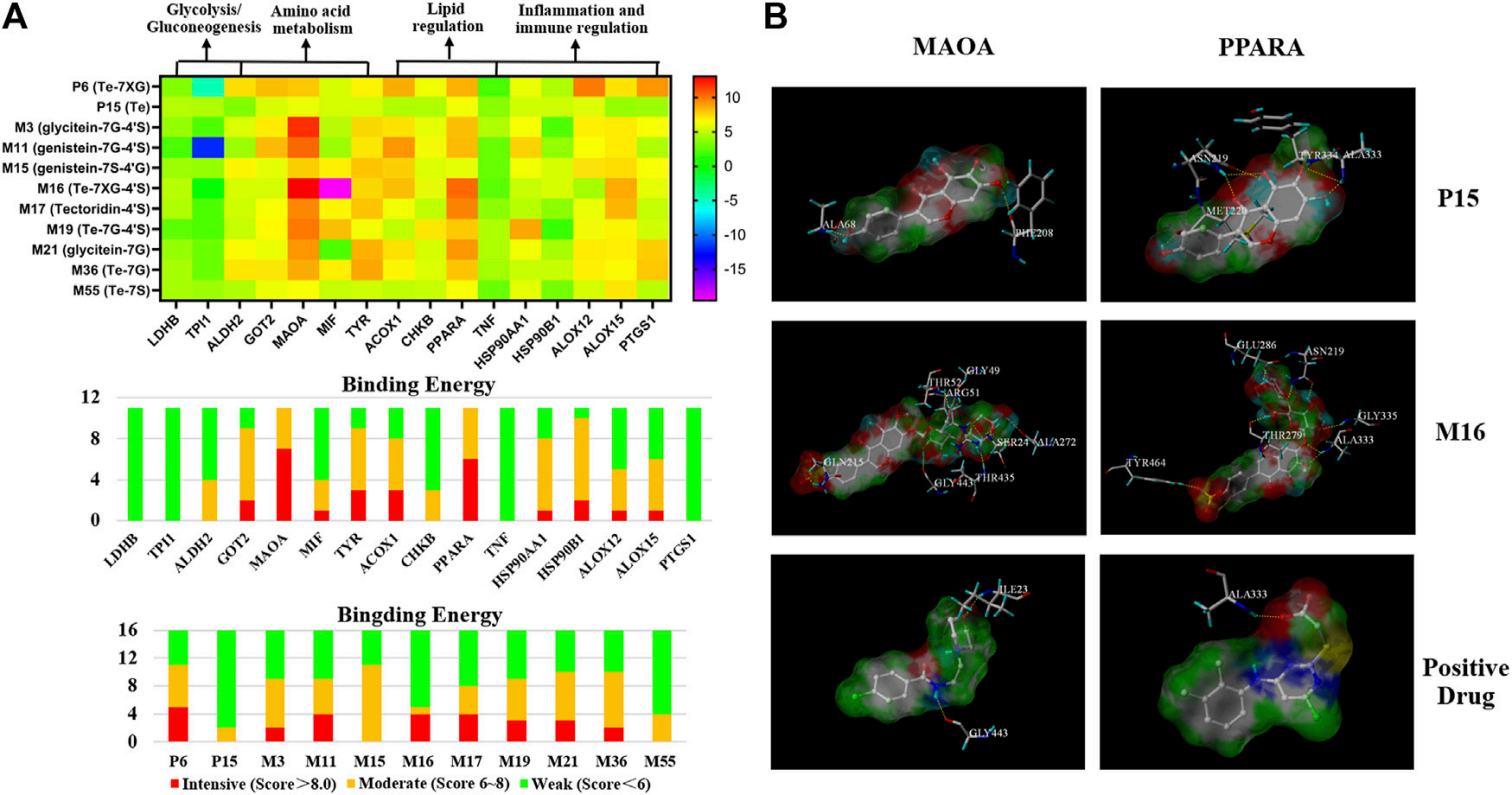
Binding interaction between the active metabolites and potential targets predicted by network analysis. **(A)** Binding energy between 11 constituents with 16 targets performed by docking. **(B)** Binding mode of tectorigenin (P15), 6″-O-xylosyltectoridin-4′-sulfate (M16), and positive drug with MAO-A and PPAR-α. (Compounds are presented with the thick stick style; hydrogen bonding interactions are expressed by yellow dotted lines. Amino acid residues which form H-bonds are presented by thin line and labeled with residue sequence.)

Based on the heat map (Figure 4A), the interactions of 11 components with 16 selective targets were ranked as intensive binding (>8.0), moderate binding (6.0–8.0), and weak binding (<6.0). Here, 8.0 was set as the cutoff value to screen the potential active components against ALD. As a result, 6″-O-xylosyltectoridin (P6) and three metabolites genistein-7-glucuronide-4′-sulfate (M11), tectoridin-4′-sulfate (M17), and 6″-O-xylosyltectoridin-4′-sulfate (M16) showed better binding ability with more than three intensive values, while weak or no inhibition effects were observed to the prototype tectorigenin (P15). As for targets, monoamine oxidase type A (MAO-A) and peroxisome proliferator–activated receptor α (PPAR-α), which rank the top two places in binding ability, contained seven and six values more than the cutoff and revealed close correlations with PF absorbed components. Among them, the interaction between MAO-A and compounds M3, M11, and M16 and PPAR-α and M16 presented the best performance. P15 and M16 were selected as representative prototype components and metabolites, respectively. Their binding mode in the active site of MAO-A and PPAR-α has been shown in a three-dimensional pattern in Figure 4B.

As for MAO-A, P15 showed two H-bond interactions with PHE208 and ALA68, which were C-7 and C-4′ phenolic hydroxyls on the A and B rings, respectively. M16 showed eight H-bond interactions as follows: four hydroxyl groups on glucose with Gly443, Gly49, ARG51, and THR52; three hydroxyl groups on xylose with THR435, SER24, and ALA27; and a phenolic hydroxyl at C-4′ on the B ring with GLN215. While Gly443 and ILE23 were involved in the N-H Bond interactions with a representative MAO-A inhibitor moclobemide, the former was also the connection site of M16 with MAO-A.

Along the similar lines, interaction between P15 and PPAR-α showed three H-bond interactions, a phenolic hydroxyl, and a carbonyl group from the isoflavonoid skeleton with ASN219; a methoxy group and a phenolic hydroxyl on the A ring with ALA333; and a methoxy group on the A ring with TYR334. Similarly, M16 showed five H-bond interactions, two phenolic hydroxyls with ASN219 and ALA333, and three hydroxyl groups on glucose and xylose with THR279, Glu286, and Gly335. In addition, the interaction of C-4′-sulfate with TYR 279 was also observed. As the positive comparison, PPAR-α agonist WY14643 shared the same residue ALA333 with P15 and M16, which was the active site for the treatment on ALD. Collectively, the docking results above showed that glycosylation or sulfation increased the binding activity comparison with the prototype.

### 3.5 Experimental validations of the pharmacological effects and the molecular mechanisms of PF against ALD

**FIGURE 5:**
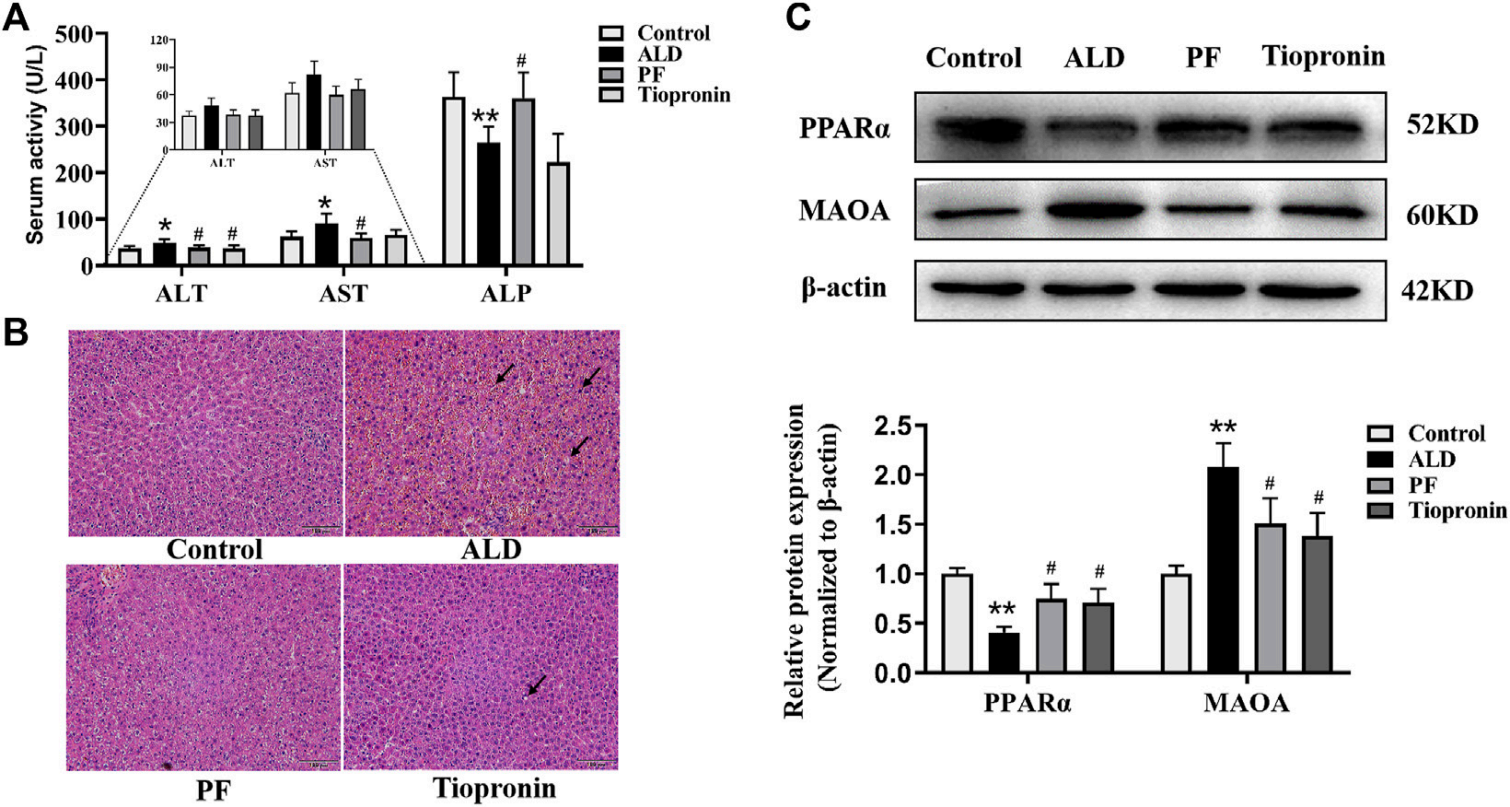
Protective effect of PF on ethanol induced rat liver injury. **(A)** Serum ALT, AST and ALP were measured using the automatic biochemistry analyzer. **(B)** Histological changes were observed using H&E staining (100×). The arrow indicates swelling and nuclear deviation, red blood cells overflow, or lipid vacuolation. **(C)** Effects of PF on PPAR-α and MAO-A protein levels in liver tissue based on the Western blotting assay. Data are presented as the mean ± SD (*n* = 3), *p < 0.05 and **p < 0.01 vs. control group. ^#^ *p* < 0.05 vs. model group.

We further verified the pharmacological effects and the prediction mechanisms of PF against ALD based on the rat model. As shown in Figure 5A, ALT and AST levels were enhanced in the ethanol-treated group in comparison with the control group whereas that of ALP decreased significantly (*p* < 0.05). Conversely, supplementation of PF could effectively reduce the activities of AST and ALT as well as increase the level of ALP (*p* < 0.05). Similarly, tiopronin reduced the content of ALT (*p* < 0.05), while there was no statistical difference in the AST and ALP activities (*p* > 0.05). H&E staining results indicated that PF and tiopronin markedly alleviated ethanol-triggered microvesicular steatosis with mild swelling and nuclear deviation and red blood cells overflow as well as certain lipid vacuolation in hepatocytes (Figure 5B).

To validate whether the targets MAO-A and PPAR-α were involved in the protective effect of ALD by PF, their protein levels in the liver tissues were examined. As shown in Figure 5C, the expression levels of MAO-A and PPAR-α protein respectively increased and decreased in the ALD model rats, compared to the normal control group (*p* < 0.01). As hypothesized, treatment with PF significantly recovered the protein expression of MAO-A and PPAR-α compared to the model group (*p* < 0.05). Notably, the effect of PF was approximately equal to that of tiopronin. The verification results are consistent with the network prediction results.

## 4 Discussion

In general, PF is given as a tea drink or compounded preparations with other herbal medicines by oral administration in the daily life or clinic therapy. Thus, exploring the constituents absorbed and excreted *in vivo* can narrow the screening scope of effective forms and build a foundation for the follow-up mechanism research of PF in treating various diseases. In the present study, a global metabolic profile of PF including the identification and classification was provided by detecting the metabolites in rat plasma, urine, bile, and feces after oral administration owing to high sensitivity of the UPLC-QTOF MS system. Moreover, the relative content of the prototypes and metabolites in the four biological samples were determined to evaluate the contribution of different metabolic reactions *in vivo*. As shown in Table 1, two categories of the parent compounds and related metabolites, including flavonoids and saponins, were observed in rat biological samples. The saponins were only detected in urine and feces in the form of prototypes or metabolites that were produced from phase I metabolism, such as hydroxylation, dehydroxylation, methylation, demethylation, and reduction. The flavonoids were generally speculated to be the bioactive components, and the effective forms should be absorbed into blood with appropriate concentrations.

If we evaluate the contribution of different metabolic reactions to the metabolite formation according to their number and relative content, we could find that sulfation and glucuronidation are both major metabolic reactions for PF flavonoids. The route of *in vivo* metabolism or biotransformation for flavonoids is a process to transform them into more hydrophilic metabolites, which not only enhanced their oral absorption and bioavailability but also enhanced the excretion from the body *via* the bile and urine. O'Leary et al. (2003) demonstrated that the isoflavone conjugates formed at intestinal are easy to be transported into the hepatocytes and then excreted into the bile. Moreover, hepatic uptake and efflux transporters on the basolateral membrane participate in drug elimination, which can facilitate endogenous compounds and metabolites with poor membrane permeability to transport into hepatocytes (Funk, 2008). Except for some conjugated metabolites that were eluted in bile, deconjugated by intestinal microflora, and excreted to feces, the others were reabsorbed *in vivo* and then underwent enterohepatic circulation. Therefore, the metabolites with higher relative content in bile were also selected for a follow-up mechanism analysis.

The network analysis combined with the docking analysis revealed that 6″-O-xylosyltectoridin (P6) and three metabolites genistein-7-glucuronide-4′-sulfate (M11), tectoridin-4′-sulfate (M17), and 6″-O-xylosyltectoridin-4′-sulfate (M16) may be the effective forms in the treatment of PF on ALD. The latter three belong to conjugated metabolites. Over the past two decades, several studies showed that glucuronides, sulfates, or bis-conjugates were the major existent form of flavonoids in systemic circulation and closely associated with their pharmacological actions. Our previous study found that tectorigenin-7S (M55) and tectorigenin-7G (M36) exhibited stronger inhibitory activity against aldose reductase than tectorigenin (P15) (Qu et al., 2014). In addition, several flavonoids conjugates, such as daidzein-7-glucuronide-4′-sulfate; daidzein-4′,7-disulfate; apigenin-7-*O*-glucuronide; quercetin-3-*O*-glucuronide; quercetin-4′-*O*-glucuronide; quercetin-3′-*O*-sulfate; and luteolin-7-*O*-glucuronide, have also been proved to possess some pharmacological activities, including anti-inflammatory (Min et al., 2009), anti-oxidative (Moon et al., 2001), antitumor (Chuang et al., 2016), and triglyceride-lowering effects (Eseberri et al., 2019). Therefore, these Phase II metabolites may be responsible for the pharmacological and medicinal properties of flavonoids *in vivo*, and the conjugation site should be considered as their benefit for structural modification.

When compare the difference in connecting targets, 6″-O-xylosyltectoridin (**P6**), 6″-O-xylosyltectoridin-4′-sulfate (M16), and tectoridin-4′-sulfate (M17) were strongly associated with fatty acid metabolism, adipocytokine signaling pathway, arginine and proline metabolism, NOD-like receptor signaling pathway, and glycolysis/gluconeogenesis *via* targeting ALDH2 and TNF. 6″-O-xylosyltectoridin (P6) was also involved in the NOD-like receptor signaling pathway and glycolysis/gluconeogenesis *via* regulating the candidate targets HSP90A1 and TPI1. Remarkably, GOT2 and CHKB, involved in the regulation of lipid and amino acid metabolism, were only found to be related with the metabolites genistein-7G-4′S (M11), 6″-O-xylosyltectoridin-4′-sulfate (M16), and tectoridin-4′S (M17). These results showed that the protective role of PF depended on the interacting and synergetic of both prototypes and metabolites. The prototypes are more likely to act on glycolysis/gluconeogenesis as well as inflammation and immune regulation, while the metabolites are highly involved in regulation of lipid and amino acid metabolism.

**FIGURE 6:**
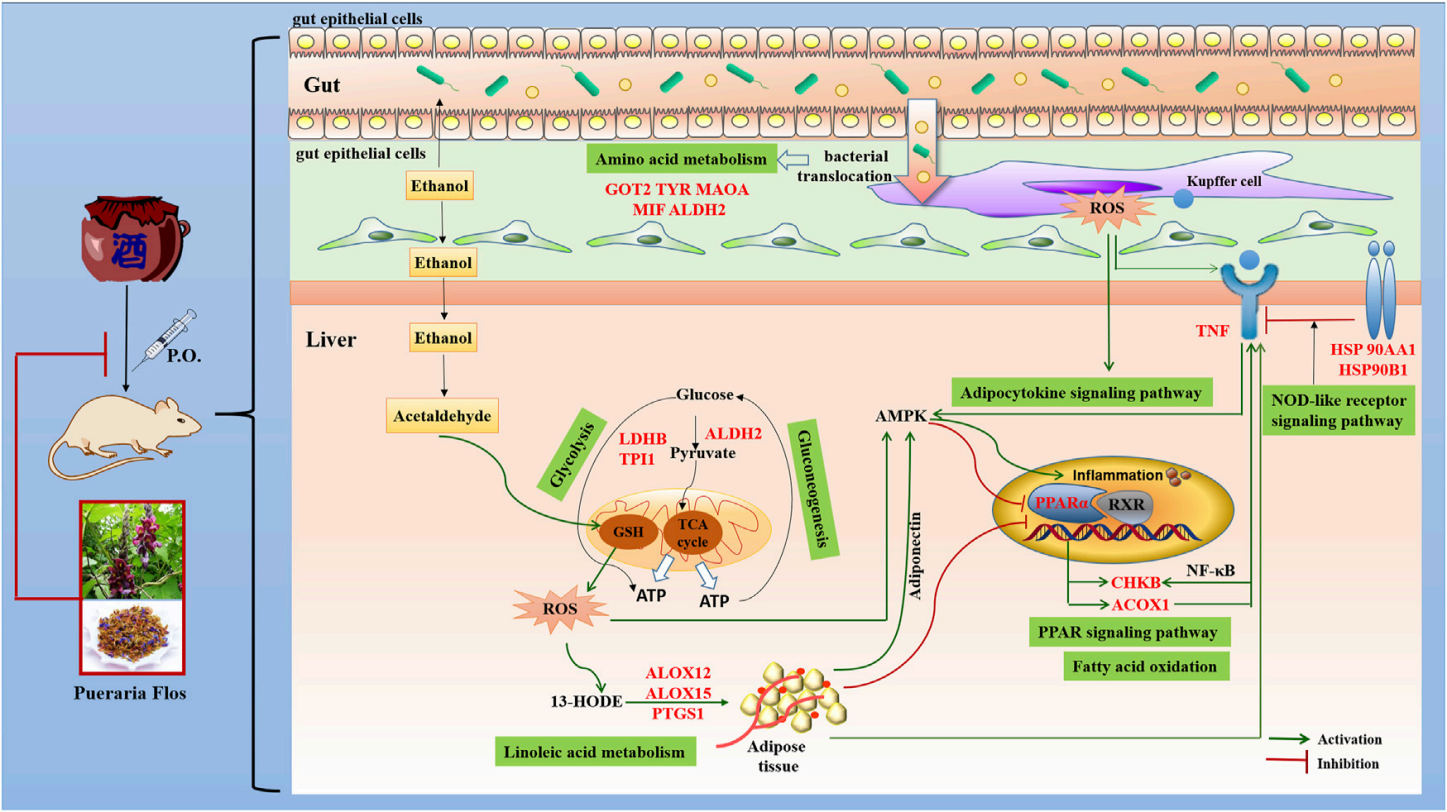
Potential molecular mechanism of PF acting on ALD.

ALD is characterized by oxidative stress, inflammation, and disturbance of hepatocyte metabolism as well as bacterial translocation (Louvet and Mathurin, 2015). In the present study, the KEGG enrichment analysis showed that the targets were regulated by 16 genes correlated with multiple biological processes inclusive of nine pathways, which were interacting and synergetic. Combined with docking results, the regulation of lipid and amino acid metabolism should highly involve in the protective effect of PF against ALD (Figure 6).

Growing evidence has demonstrated that the development of ALD is associated with disturbance of lipid regulation. Fatty acid metabolism (hsa00071), adipocytokine signaling pathway (hsa04920), and PPAR signaling pathway (hsa03320), selected on the basis of the enrichment by five genes ACOX1, CHKB, ALDH2, PPARA, and TNF, participate in the lipid regulation process of PF. Peroxisome proliferator–activated receptor α (PPAR-α), a key nuclear transcription factor abundantly expressed in the liver, plays a major role in lipid metabolism regulation. Several studies reported that PPAR-α agonists were expected to be a treatment for ALD by reducing oxidative stress, regulating fatty acid synthesis and oxidation, inhibiting the production and release of inflammatory and profibrogenic factors, and alleviating liver tissue damage (Kersten, 2014). As the target genes of PPAR-α, the expression of peroxisomal acyl-coenzyme A oxidase 1 (ACOX1) and choline/ethanolamine kinase (CHKB) through fatty acid oxidation will be reduced, and the accumulation of triglyceride in the liver and the content of TNF-α in serum was accordingly increased in the development and progression of ALD. Concordantly, increased TNF affects the expression of CHKB through the NF-kB pathway and induces inflammation, which aggravates the course of disease.

Abnormal amino acid metabolism, including tyrosine metabolism (hsa00350), phenylalanine metabolism (hsa00360), and arginine and proline metabolism (hsa00330), is closely related to intestinal flora metabolism, and bacterial translocation from the gut microbiota into the portal blood stream is an important driver of ALD (Vassallo et al., 2015). MAO-A, a mitochondrial enzyme, exists mainly in the human liver, kidney, small intestine, and nerve tissues and could remove the metabolites of tyrosine and phenylalanine by oxidative deamination. It had been closely associated with neurological and psychiatric disorders due to its degradation of catecholamine neurotransmitter. Recent studies provide insights into the application of MAO-A as a novel predictor of clinical outcomes that MAO-A expression was negatively correlated with the alcohol consumption level and hepatocellular carcinoma (Cervera-Juanes et al., 2016). Increasing MAO-A expression or enzyme activity may be a new approach that can be used for ALD treatment. In addition, researchers founded that ALDH2, aldehyde dehydrogenase located in mitochondria, was the downstream gene of MAO-A. A link between capacity to reduce alcohol consumption and increased liver ratio of MAO to ALDH2 has been established by using an animal model (Cervera-Juanes et al., 2016). Other targets, such as GOT2, TYR, and MIF, are also involved in the gut bacterial pathway for dopamine and tyrosine metabolism, which affect the intestinal permeability and bacterial translocation.

An increasing number of studies have shown that inflammation and immune regulation was also critical for the progression of ALD. Heat shock protein 90 (hsp90), an emerging therapeutic target in ALD, was a main effector in the NOD-like receptor signaling pathway (hsa04621). It is involved in initiation of the early phases of ER stress contributing to stimulation and accumulation of hepatic lipids (Ambade et al., 2014). Hsp90 inhibitors could alleviate serum ALT, endotoxin, and pro-inflammatory cytokines such as TNF-α in acute and chronic alcoholic liver injury and regulating PPAR-α to influence fatty acid oxidation and synthesis. In addition, ALOX12, ALOX15, and PTGS1, which catalyze the generation of leukotrienes and prostaglandins by arachidonic acid metabolism (hsa00590), also participate in the processes of ALD through activation of inflammatory responses (Zhang et al., 2017). Their chemical inhibitors have been confirmed to significantly alleviate alcohol-induced oxidative stress, lipid accumulation, and liver damage. In addition, LDHB, TPI1, and ALDH2, which act as master regulatory genes of glycolysis/gluconeogenesis (hsa00010) due to the increased cells’ need for oxygen by chronic alcohol consumption, also play a vital role in the treatment of PF in ALD.

In this study, the bioinformatic method was combined to elucidate the active components and mechanism of PF in the treatment of ALD. Some of the predicted results are confirmed by *in vivo* experiments, which preliminarily prove the scientific nature of this method. In addition, some of the compound–target–pathway interactions predicted by the network analysis have also been confirmed in the previous studies. For example, protective effects of genistein against chronic alcohol-induced liver injury in mice were related to regulate expression of inflammatory-related factors TNFα, NF-κB, and PTGS1 (Zhao et al., 2016). Tectoridin, a characteristic isoflavone glycoside found in PF and *Belamcanda* chinensis (L.) DC., could protect against ethanol-induced liver steatosis mainly by modulating the disturbance of the PPAR-α pathway and ameliorating the mitochondrial function (Xiong et al., 2010). In addition, attenuated alcoholism by daidzin has been proved to be associated with the liver mitochondrial MAO–ALDH2 pathway (Ambade et al., 2014). However, other constituents and targets especially conjugated metabolites predicted in the present study still need to be validated in the follow-up study.

There are still two shortcomings in this study. On the one hand, benchwork assessing affinity using several technologies such as the surface plasmon resonance biosensor should be combined with a docking analysis to evaluate and validate the importance of relative compounds and targets. As our research focus on the *in vivo* metabolites, the phase II metabolites including glucuronides, sulfates, and/or bis-conjugates that account for the most part were difficult to isolate due to their exclusivity distribution in the biological matrix rather than the plant kingdom. Therefore, the absence of metabolite references restricted further affinity evaluation. On the other side, the single dose in the present study was chosen to continue and compare with our previous metabolism research, which determined the pharmacokinetic parameters of tectoridin and tectorigenin after oral administration at dosages of 200 and 130 mg/kg, respectively (Qu et al., 2012; Wang S. et al., 2013). However, three dosages (low, middle, and high) should be set to reflect the dose–effect relationship more scientifically in the pharmacological research. These deficiencies will be improved in our subsequent studies.

## 5 Conclusion

**FIGURE 7:**
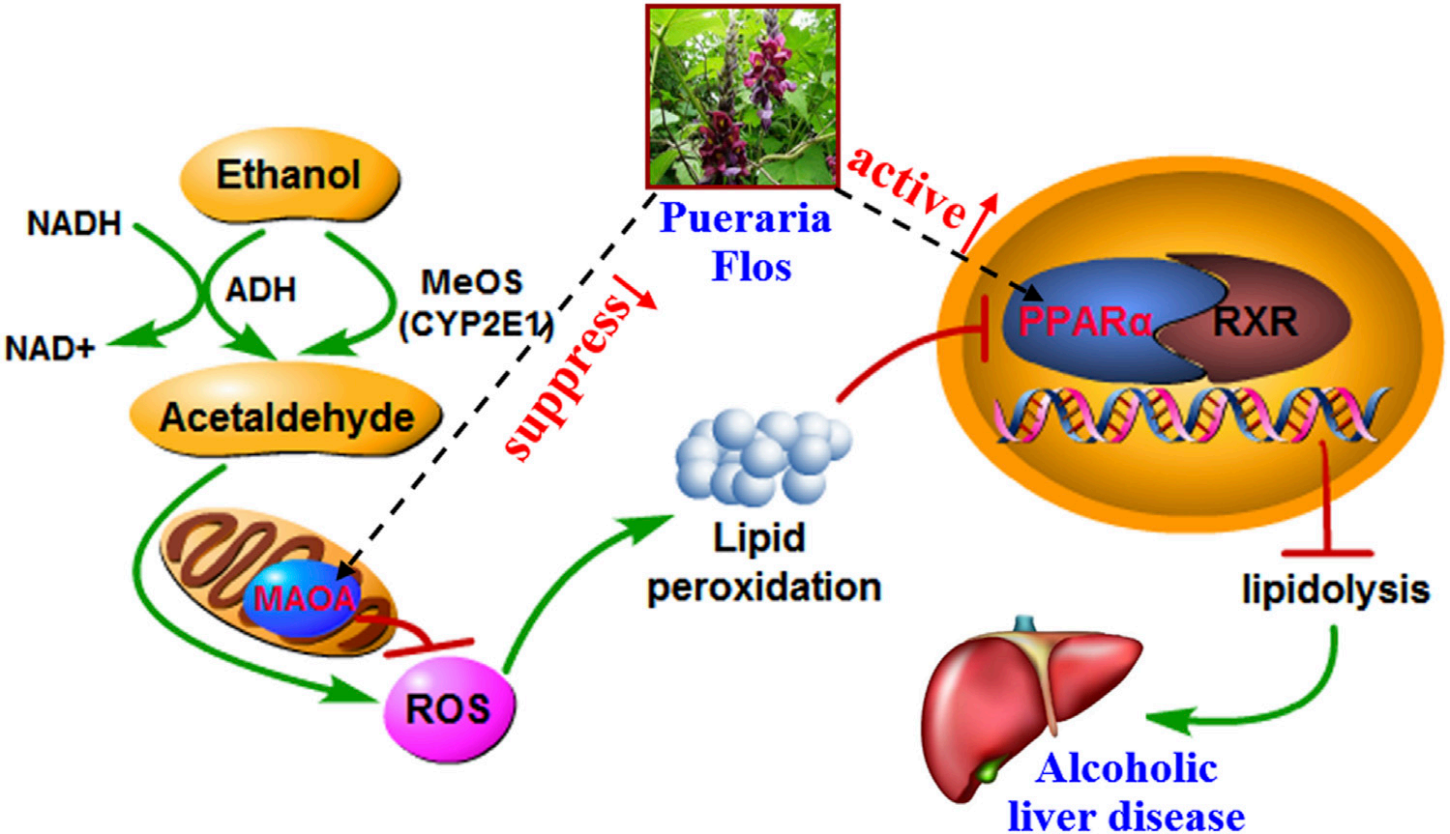
Overview of potential mechanisms underlying the protective effects of PF on alcohol-induced liver injury.

The present study has developed a sensitive and rapid method for the separation and identification of the absorbed constituents and metabolites of PF *in vivo* for the first time. Glucuronidation, sulfation, methylation, hydroxylation, and reduction are the major metabolic reactions. Furthermore, the constructed absorbed constituent–target–pathway–disease network and the docking analysis revealed that phase II metabolites may play more important roles in the PF-mediated protection against ALD. Also, the protective effects and predicted mechanism associated with decreased and elevated expression of MAO-A and PPAR-α in rat ALD models were also validated (Figure 7). However, the absence of metabolite references restricted the reliability of predicted conclusions about importance of key metabolites. In the future, we will try to purify the glucuronides, sulfates, and/or bis-conjugated metabolites and employed the benchwork assay to obtain the affinity and Michaelis constant of active compounds bound to key targets, which make the research more credible.

## Data Availability

The datasets presented in this study can be found in online repositories. The names of the repository/repositories and accession number(s) can be found in the article/Supplementary Material.

## Supplementary material

Supplementary Figure S1 Representative base peak chromatogram (BPC) of seven mixed reference compounds **(A)** and PF extract **(B)** in the negative ion mode. See Supplementary Table 1 for the peak numbers, and see section 2.4 UPLC-QTOF MS analysis for UPLC-QTOF MS conditions.

Supplementary Figure S2 Extracted ion chromatograms of all identified metabolites in blank biosamples determined by UPLC-QTOF MS analysis.

Supplementary Table S1 Compounds identified from the flower of *Pueraria montana* var. *thomsonii* (Benth.) M.R. Almeida extract by the UPLC-QTOF/MS method.

Supplementary Table S2 Potential targets of 13 absorbed constituents from Puerariae Flos.

Supplementary Table S3 Therapeutic targets of alcoholic fatty liver.

## Abbreviations

ACOX1, peroxisomal acyl-coenzyme A oxidase 1; ALDH2, aldehyde dehydrogenase; ALOX12, polyunsaturated fatty acid lipoxygenase ALOX12; ALOX15, polyunsaturated fatty acid lipoxygenase ALOX15; CHKB, choline/ethanolamine kinase; GOT2, aspartate aminotransferase; HSP90AA1, heat shock protein HSP 90-alpha; HSP90B1, endoplasmin; LDHB, L-lactate dehydrogenase B chain; MAO-A, amine oxidase (flavin-containing) A; MIF, macrophage migration inhibitory factor; PPAR-α, peroxisome proliferator–activated receptor alpha; PTGS1, prostaglandin G/H synthase 1; TNF, tumor necrosis factor; TPI1, triosephosphate isomerase; TYR, tyrosinase; UPLC, ultra-performance liquid chromatography.
