# Supplementary material for: Systematic characterization of Puerariae Flos metabolites in vivo and assessment of its protective mechanisms against alcoholic liver injury in a rat model
Source: Front Pharmacol. 2022 Aug 30;13:915535. doi: 10.3389/fphar.2022.915535 (PMC9468746; doi:10.3389/fphar.2022.915535)
Supplement: Supplementary file 3 [file Table1.DOCX]

**Supplemental Table 1 Compounds identified from the flower of *Pueraria thomsonii* Benth extract by UPLC-QTOF/MS method**

No. t_R_ Identification Elemental [M-H]^—^and fragments

(min) composition Observed Calculated Error

mass (Da) mass (Da) (ppm)

1 4.44 puerarin C_21_H_19_O_9_ 415.1028 415.1029 -0.2

C_14_H_7_O_3_ 223.0401 223.0395 2.7

2 4.66 dihydrokaempferol-7-O-glucoside C_21_H_21_O_11_ 449.1064 449.1084 -4.5

C_15_H_11_O_6_ 287.0547 287.0556 -3.1

C_14_H_11_O_5_ 269.0471 269.045 7.8

3 5.11 6-hydroxygenistein-6,7-di-O-glucoside C_27_H_29_O_16_ 609.1471 609.1456 2.5

C_21_H_19_O_11_ 447.0918 447.0927 -2.0

C_15_H_9_O_6_ 285.0404 285.0399 1.8

4 5.31 nicotiflorin C_27_H_29_O_15_ 593.151 593.1506 0.7

C_15_H_9_O_6_ 285.038 285.0399 -6.7

C_7_H_3_O_4_ 151.0027 151.0031 -2.6

5 6.43 daidzin C_21_H_19_O_9_ 415.0999 415.1029 -7.2

C_15_H_9_O_4_ 253.048 253.0501 -8.3

6 6.73 glycitein-7-O-xylosylglucoside C_27_H_29_O_14_ 577.1542 577.1557 -2.6

C_22_H_21_O_10_ 445.1094 445.1135 -9.2

C_16_H_11_O_5_ 283.0602 283.0606 -1.4

C_15_H_8_O_5_ 268.037 268.0372 -0.7

C_14_H_8_O_4_ 240.0414 240.0423 -3.7

7 7.27 glycitin C_22_H_21_O_10_ 445.1129 445.1135 -1.3

C_16_H_11_O_5_ 283.0608 283.0606 0.7

C_15_H_8_O_5_ 268.0365 268.0372 -2.6

C_14_H_7_O_4_ 239.0341 239.0344 -1.3

8 9.05 rutin C_27_H_29_O_16_ 609.1462 609.1456 1.0

C_15_H_9_O_7_ 301.0331 301.0348 -5.6

C_14_H_7_O_6_ 285.0399 285.0399 0.0

9 9.73 genistin C_21_H_19_O_10_ 431.098 431.0978 0.5

C_15_H_9_O_5_ 269.0451 269.045 0.4

C_8_H_5_O_2_ 133.0302 133.029 9.0

10 ^a)^ 9.84 tectorigenin-7-O-β-D-xylosylglucoside C_27_H_29_O_15_ 593.1509 593.1506 0.5

C_16_H_11_O_6_ 299.0565 299.0556 3.0

C_15_H_8_O_6_ 284.0326 284.0321 1.8

C_14_H_7_O_5_ 255.0302 255.0293 3.5

11 10.09 biochanin A-7-O-β-D-glucopyranoside C_22_H_21_O_10_ 445.1151 445.1135 3.6

C_16_H_11_O_5_ 283.0603 283.0606 -1.1

C_15_H_8_O_5_ 268.0398 268.0372 9.7

12 ^a)^ 10.62 tectorodin C_22_H_21_O_11_ 461.1097 461.1084 2.8

C_16_H_11_O_6_ 299.0561 299.0556 1.7

C_15_H_8_O_6_ 284.0322 284.0321 0.4

C_14_H_7_O_5_ 255.0302 255.0293 3.5

**Supplemental Table 1 Compounds identified from the flower of *Pueraria thomsonii* Benth extract by UPLC-QTOF/MS method (*Continued*)**

No. t_R_ Identification Elemental [M-H]^—^and fragments

(min) composition Observed Calculated Error

mass (Da) mass (Da) (ppm)

13 12.43 6-hydroxygenistein-7-O-glucoside C_21_H_19_O_11_ 447.0914 447.0927 -2.9

C_15_H_9_O_6_ 285.0414 285.0399 5.3

C_15_H_7_O_5_ 267.0268 267.0293 -9.4

14 12.54 apigenin-4'-O-glucoside C_21_H_19_O_10_ 431.0979 431.0978 0.2

C_15_H_9_O_5_ 269.0434 269.045 -5.9

C_7_H_3_O_4_ 151.0027 151.0031 -2.6

15 14.84 6-hydroxybiochanin A-6,7-di-O-glucoside C_28_H_31_O_16_ 623.1611 623.1612 -0.2

C_22_H_21_O_11_ 461.1045 461.1084 -8.5

C_16_H_11_O_6_ 299.056 299.0556 1.3

C_15_H_8_O_6_ 284.0301 284.0321 -7.0

16 ^a)^ 16.17 daidzein C_15_H_9_O_4_ 253.0508 253.0501 2.8

C_14_H_7_O_3_ 223.0399 223.0395 1.8

17 17.34 isomer of tectorigenin C_16_H_11_O_6_ 299.0552 299.0556 -1.3

C_15_H_8_O_6_ 284.0302 284.0321 -6.7

C_14_H_7_O_5_ 255.0313 255.0293 7.8

18 18.42 irilin D C_16_H_11_O_7_ 315.0511 315.0505 1.9

C_15_H_8_O_7_ 300.0245 300.027 -8.3

19 19 glycitein C_16_H_11_O_5_ 283.0606 283.0606 0.0

C_15_H_8_O_5_ 268.0355 268.0372 -6.3

20 ^a)^ 19.44 luteolin C_15_H_9_O_6_ 285.0402 285.0399 1.1

C_8_H_5_O_2_ 133.0294 133.029 3.0

21 20.13 vanillic acid C_8_H_7_O_4_ 167.0347 167.0344 1.8

C_6_H_4_O_2_ 108.0204 108.0211 -6.5

22 21.19 kakkalide C_28_H_31_O_15_ 607.1669 607.1663 1.0

C_17_H_13_O_6_ 313.0708 313.0712 -1.3

C_16_H_10_O_6_ 298.0479 298.0477 0.7

C_15_H_7_O_6_ 283.0257 283.0243 4.9

23 ^a)^ 21.43 genistein C_15_H_9_O_5_ 269.0453 269.045 1.1

C_8_H_5_O_2_ 133.0297 133.029 5.3

24 21.74 apigenin C_15_H_9_O_5_ 269.045 269.045 0.0

C_7_H_3_O_4_ 151.0039 151.0031 5.3

25 ^a)^ 21.87 tectorigenin C_16_H_11_O_6_ 299.0566 299.0556 3.3

C_15_H_8_O_6_ 284.0314 284.0321 -2.5

C_14_H_7_O_5_ 255.029 255.0293 -1.2

26 22.07 irisolidone-7-O-β-D-glucopyranoside C_23_H_23_O_11_ 475.124 475.124 0.0

C_17_H_13_O_6_ 313.0683 313.0712 -9.3

C_16_H_10_O_6_ 298.0464 298.0477 -4.4

C_15_H_7_O_6_ 283.0221 283.0243 -7.8

27 22.42 iristectorigenin A C_17_H_13_O_7_ 329.0664 329.0661 0.9

C_16_H_10_O_7_ 314.0397 314.0427 -9.6

**Supplemental Table 1 Compounds identified from the flower of *Pueraria thomsonii* Benth extract by UPLC-QTOF/MS method (*Continued*)**

No. t_R_ Identification Elemental [M-H]^—^and fragments

(min) composition Observed Calculated Error

mass (Da) mass (Da) (ppm)

28 23.6 formononetin C_16_H_11_O_4_ 267.0648 267.0657 -3.4

C_15_H_8_O_4_ 252.0423 252.0423 0.0

29 25.47 astragaloside VIII C_47_H_75_O_17_ 911.4992 911.5004 -1.3

C_41_H_65_O_13_ 765.4435 765.4425 1.3

C_36_H_55_O_8_ 615.3893 615.3897 -0.6

C_30_H_49_O_3_ 457.3675 457.3682 -1.5

30 25.53 soyasaponin I C_48_H_77_O_18_ 941.507 941.511 -4.2

C_42_H_67_O_14_ 795.4492 795.4531 -4.9

C_36_H_55_O_8_ 615.3887 615.3897 -1.6

C_30_H_49_O_3_ 457.3661 457.3682 -4.6

31 25.85 soyasaponin II C_47_H_75_O_17_ 911.4987 911.5004 -1.9

C_41_H_65_O_13_ 765.4442 765.4425 2.2

C_36_H_55_O_8_ 615.3839 615.3897 -9.4

C_30_H_49_O_3_ 457.3704 457.3682 4.8

32 ^a)^ 25.9 irisolidone C_17_H_13_O_6_ 313.0717 313.0712 1.6

C_16_H_10_O_6_ 298.0481 298.0477 1.3

C_15_H_7_O_6_ 283.0242 283.0243 -0.4

C_14_H_7_O_5_ 255.0296 255.0293 1.2

33 26.01 kaikasaponin III C_48_H_77_O_17_ 925.5164 925.5161 0.3

C_42_H_67_O_13_ 779.4632 779.4582 6.4

C_36_H_57_O_8_ 617.4084 617.4053 5.0

C_36_H_55_O_7_ 599.396 599.3948 2.0

C_30_H_49_O_2_ 441.3737 441.3733 0.9

34 26.08 soyasaponin IV C_41_H_65_O_13_ 765.4414 765.4425 -1.4

C_36_H_55_O_8_ 615.3888 615.3897 -1.5

C_30_H_49_O_3_ 457.3653 457.3682 -6.3

35 26.15 kaikasaponin II C_48_H_77_O_17_ 925.5151 925.5161 -1.1

C_42_H_67_O_13_ 779.4576 779.4582 -0.8

C_36_H_57_O_8_ 617.4095 617.4053 6.8

C_36_H_55_O_7_ 599.39 599.3948 -8.0

C_30_H_49_O_2_ 441.3752 441.3733 4.3

36 26.56 kaikasaponin I C_42_H_67_O_13_ 779.4582 779.4582 0.0

C_36_H_57_O_8_ 617.3998 617.4053 -8.9

C_36_H_55_O_7_ 599.3998 599.3948 8.3

37 26.67 azukisaponin I C_42_H_67_O_13_ 779.4568 779.4582 -1.8

C_36_H_57_O_8_ 617.4074 617.4053 3.4

C_36_H_55_O_7_ 599.3961 599.3948 2.2

38 26.67 kakkasapnin I C_47_H_75_O_16_ 895.5026 895.5055 -3.2

C_41_H_65_O_12_ 749.4518 749.4476 5.6

C_36_H_55_O_7_ 599.3931 599.3948 -2.8

C_30_H_49_O_2_ 441.3705 441.3733 -6.3

**Supplemental Table 1 Compounds identified from the flower of *Pueraria thomsonii* Benth extract by UPLC-QTOF/MS method (*Continued*)**

No. t_R_ Identification Elemental [M-H]^—^and fragments

(min) composition Observed Calculated Error

mass (Da) mass (Da) (ppm)

39 26.75 baptisiasaponin I C_47_H_75_O_16_ 895.5039 895.5055 -1.8

C_41_H_65_O_12_ 749.4473 749.4476 -0.4

C_36_H_55_O_7_ 599.3946 599.3948 -0.3

C_30_H_49_O_2_ 441.3709 441.3733 -5.4

40 26.99 phaseoside IV C_48_H_75_O_17_ 923.4988 923.5004 -1.7

C_42_H_65_O_13_ 777.4481 777.4425 7.2

C_36_H_53_O_7_ 597.376 597.3791 -5.2

C_30_H_47_O_2_ 439.3568 439.3576 -1.8

41 27.55 kakkasapnin II C_42_H_65_O_13_ 777.4464 777.4425 5.0

C_36_H_53_O_7_ 597.381 597.3791 3.2

C_30_H_47_O_2_ 439.3612 439.3576 8.2

42 27.66 kakkasapnin III C_47_H_73_O_16_ 893.4911 893.4899 1.3

C_41_H_63_O_12_ 747.4379 747.432 7.9

C_36_H_53_O_7_ 597.3791 597.3791 0.0

C_30_H_47_O_2_ 439.3615 439.3576 8.9

43 28.91 sophoradiol monoglucuronide C_36_H_57_O_8_ 617.4033 617.4053 -3.2

C_30_H_49_O_2_ 441.3762 441.3733 6.6

^a)^ Compositions identified with reference compounds comparison
