# Supplementary figures and images for: Systematic characterization of Puerariae Flos metabolites in vivo and assessment of its protective mechanisms against alcoholic liver injury in a rat model
Source: Front Pharmacol. 2022 Aug 30;13:915535. doi: 10.3389/fphar.2022.915535 (PMC9468746; doi:10.3389/fphar.2022.915535)

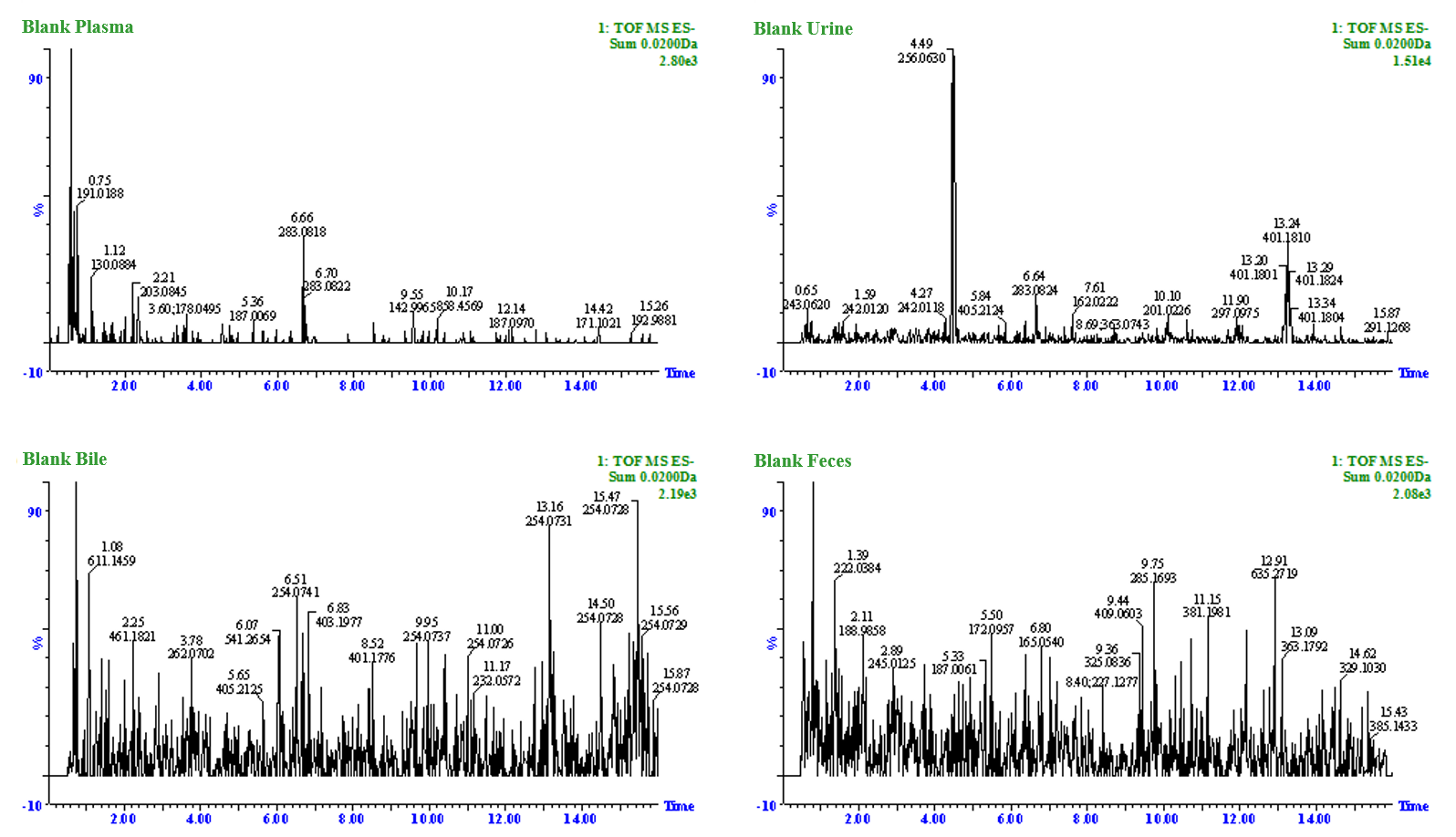

Supplement: Supplementary file 4 [file Image2.TIF]

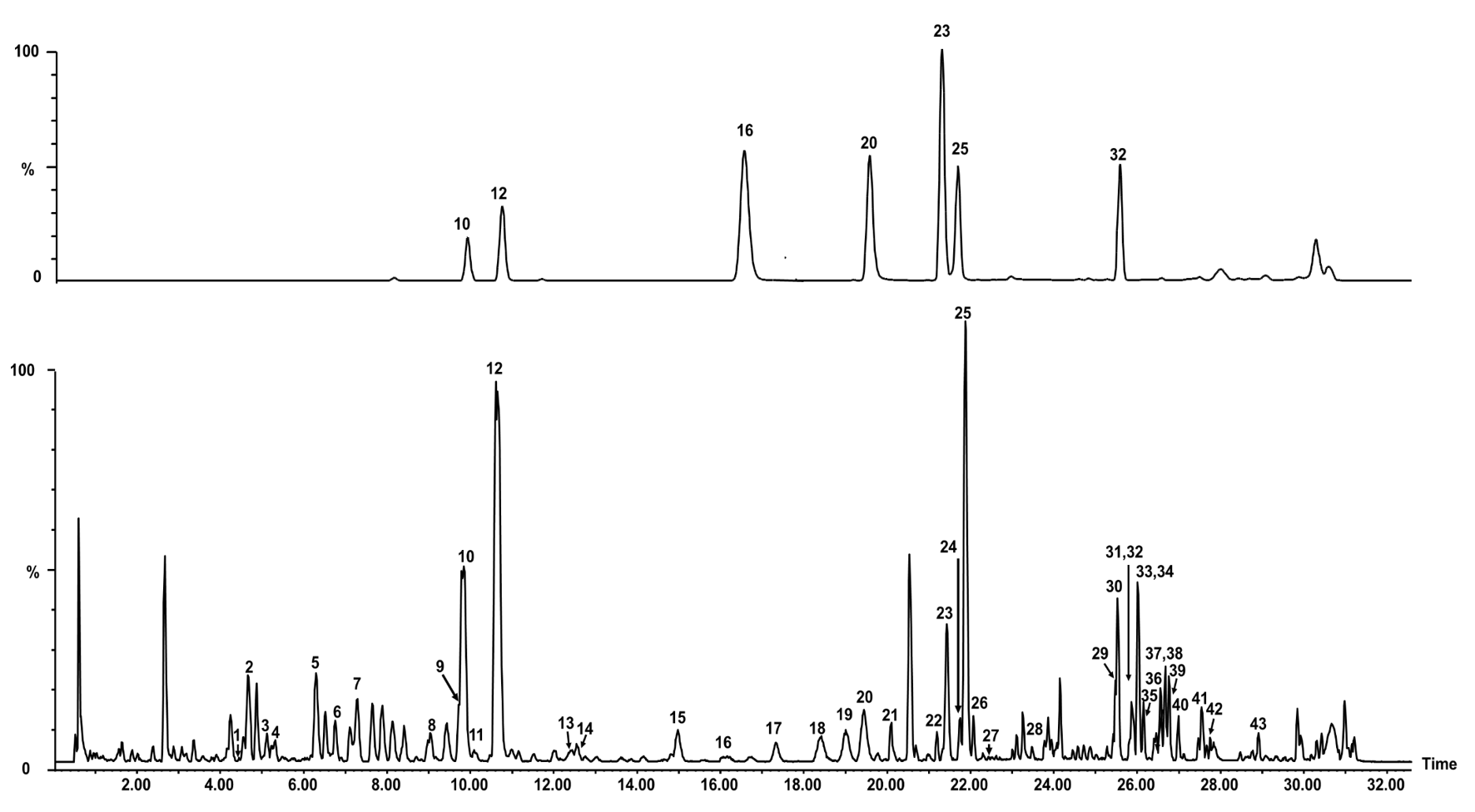

Supplement: Supplementary file 5 [file Image1.TIF]
