# Supplementary material for: Systematic characterization of Puerariae Flos metabolites in vivo and assessment of its protective mechanisms against alcoholic liver injury in a rat model
Source: Front Pharmacol. 2022 Aug 30;13:915535. doi: 10.3389/fphar.2022.915535 (PMC9468746; doi:10.3389/fphar.2022.915535)
Supplement: Supplementary file 9 [file Table4.DOCX]

**Pathological picture**

**（1）Control**

**
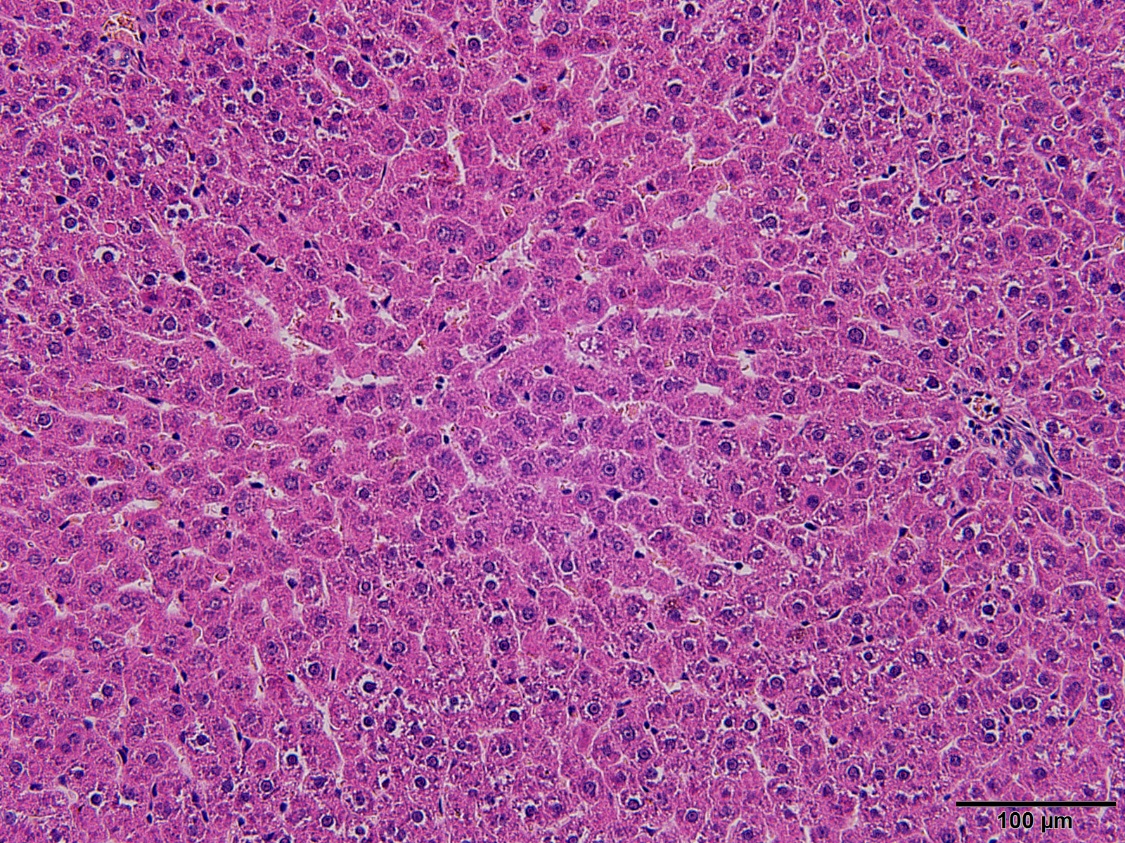
**

**（2）ALD**

**
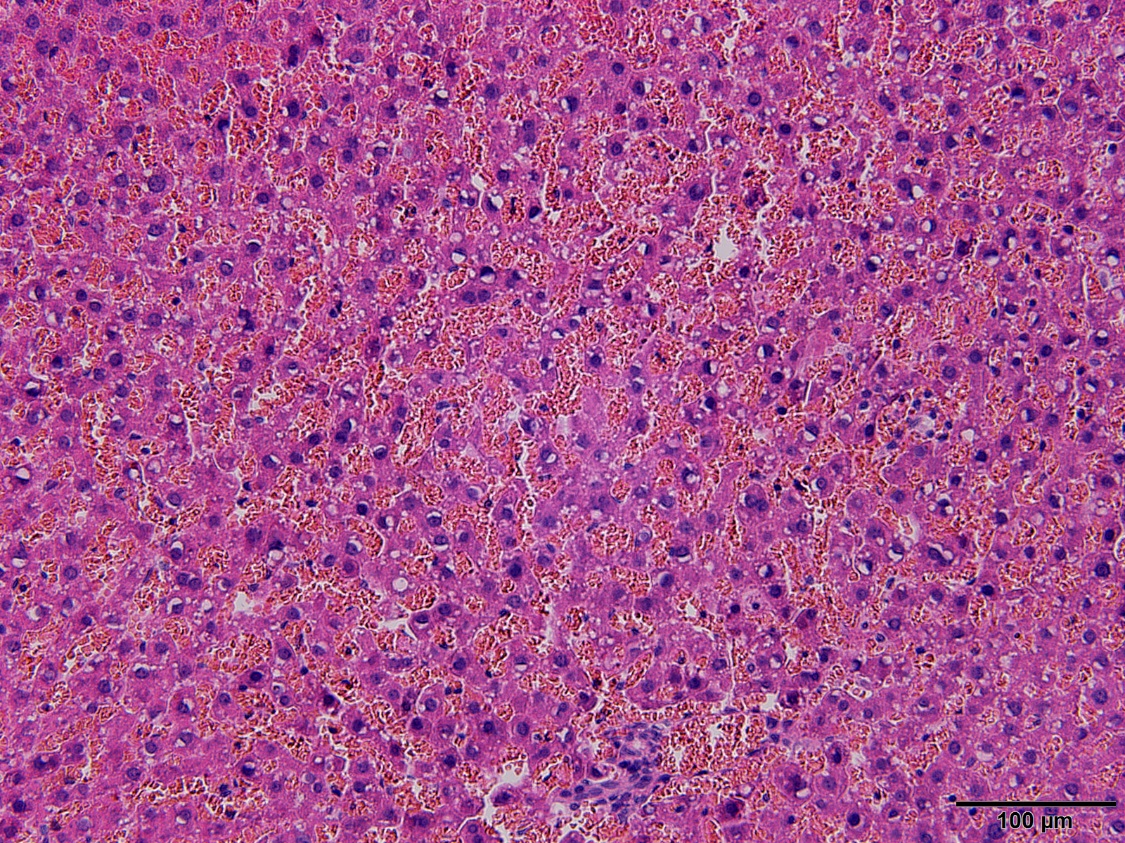
**

**（3）PF**

**
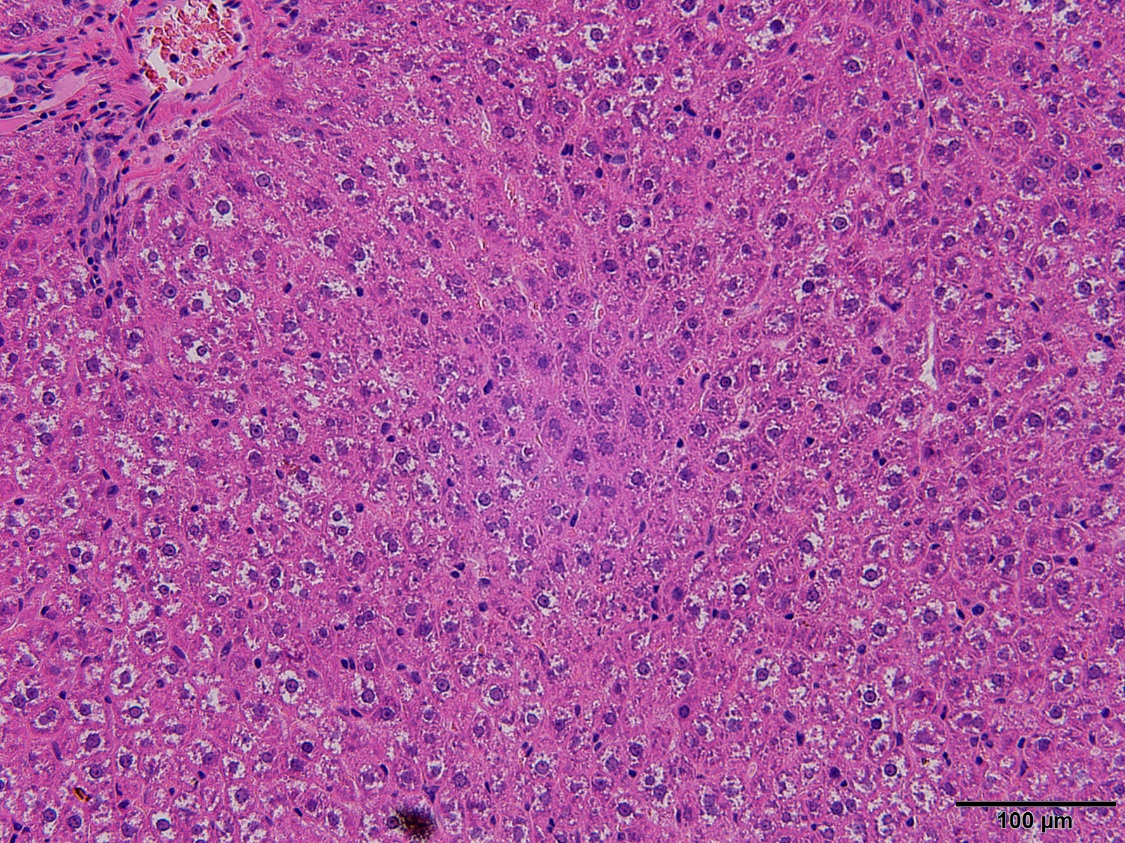
**

**（4）Tiopronin**

**
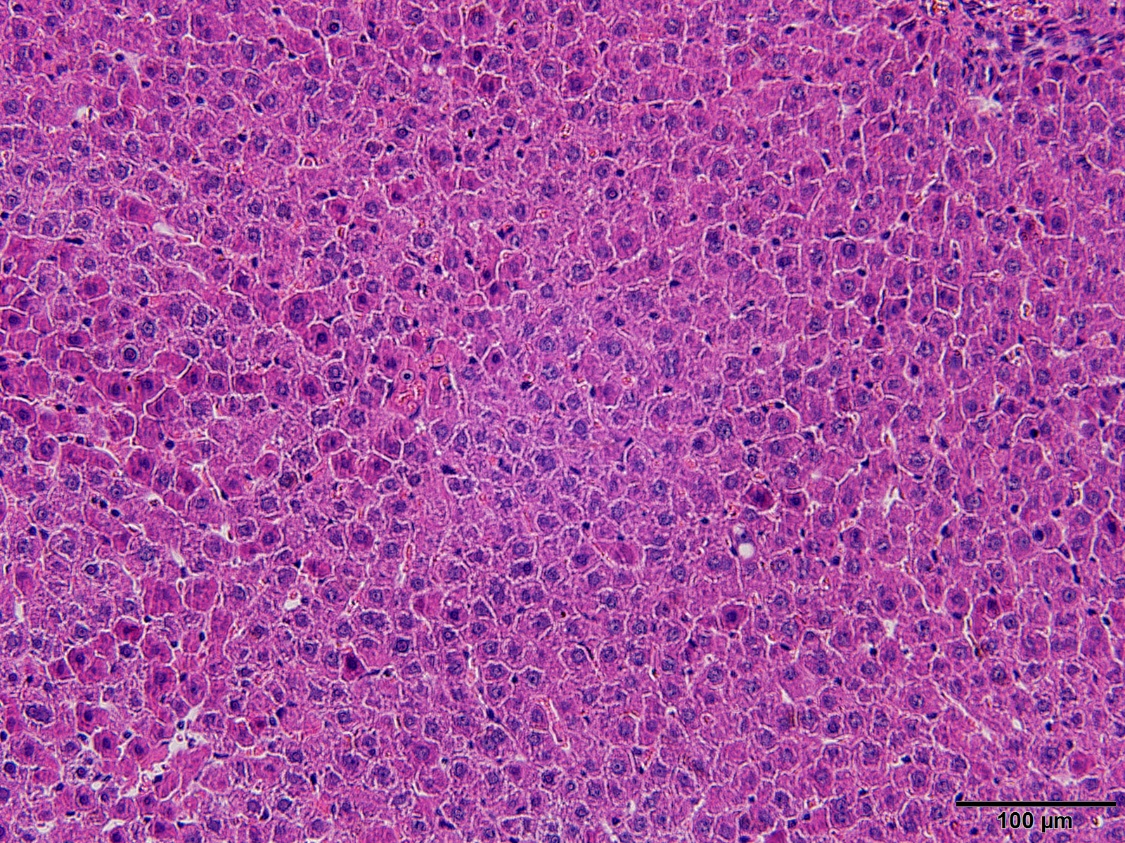
**

**Western-Blot bands**

**一、MAOA**

**（1）MAOA-1**

**
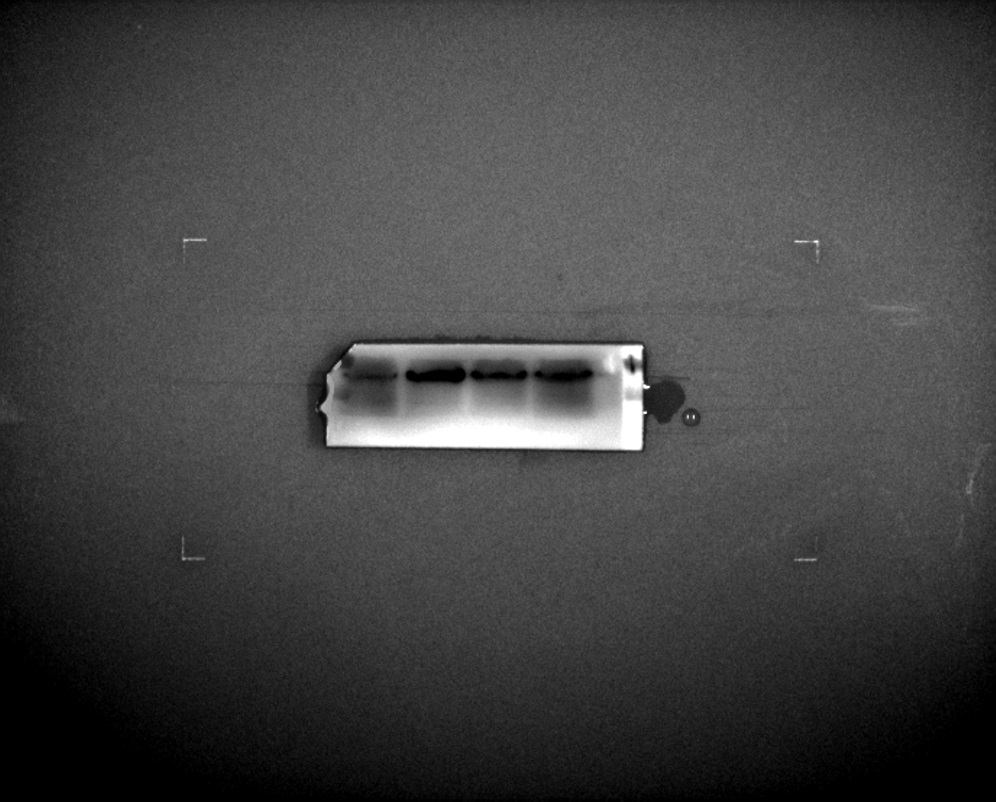
**

**（2）MAOA-β-actin-1**

**
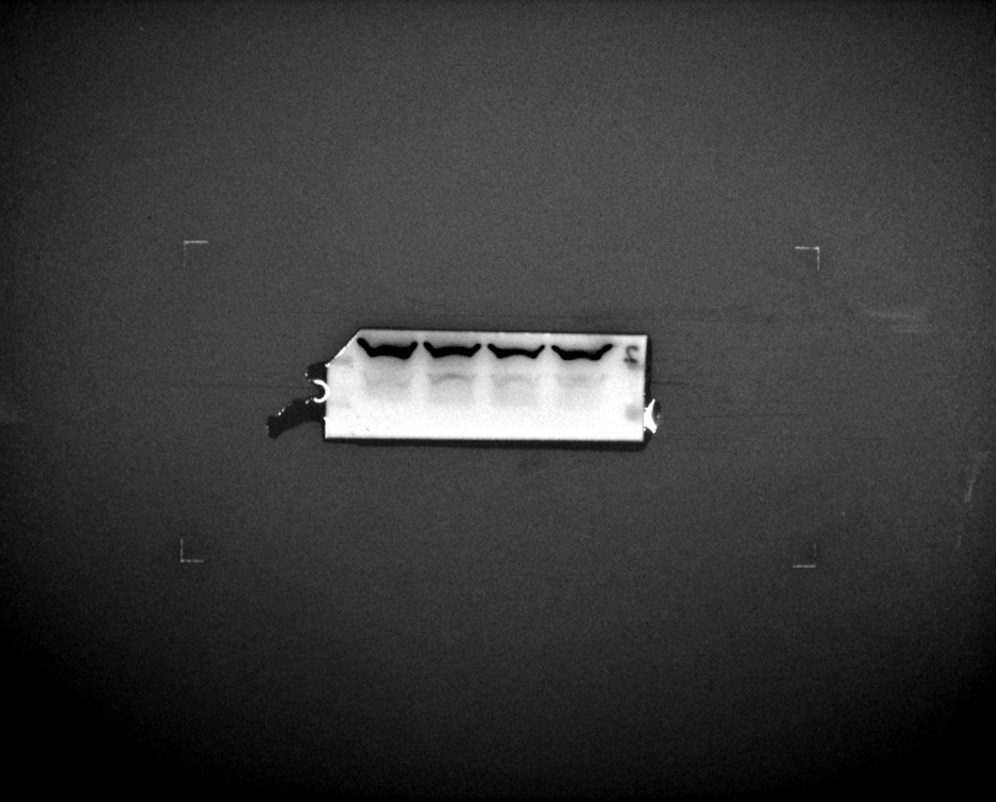
**

**（3）MAOA-2**

**
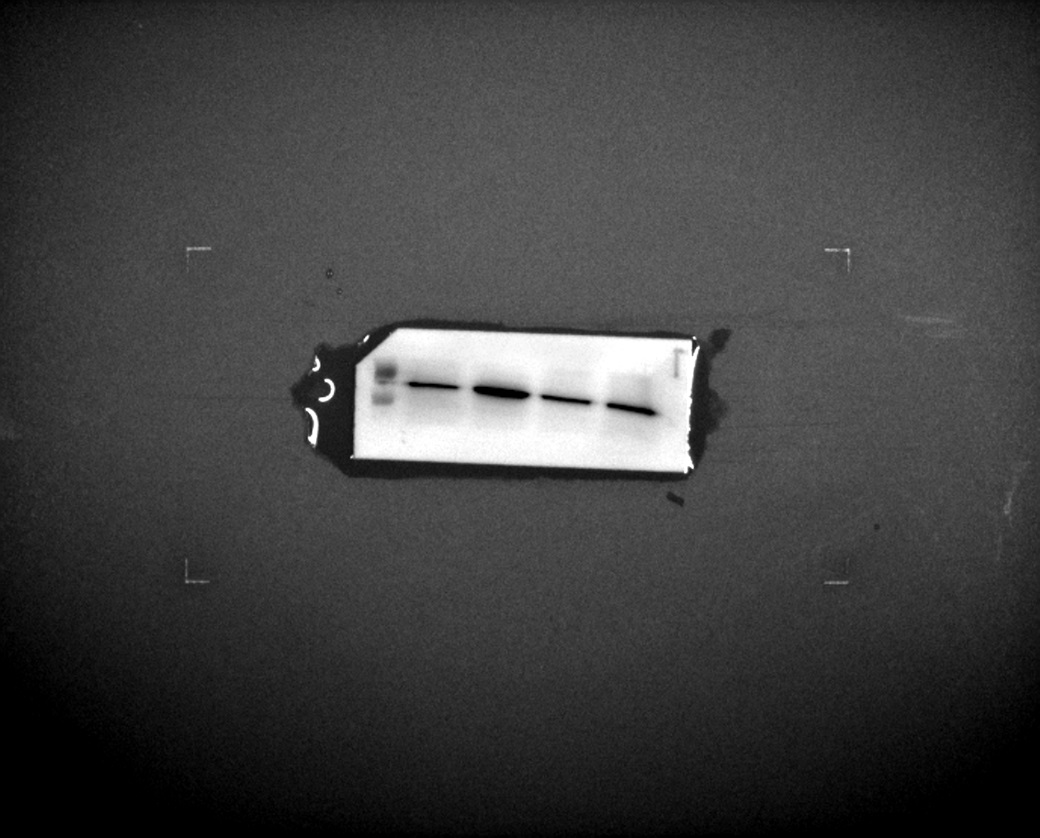
**

**（4）MAOA-β-actin-2**

**
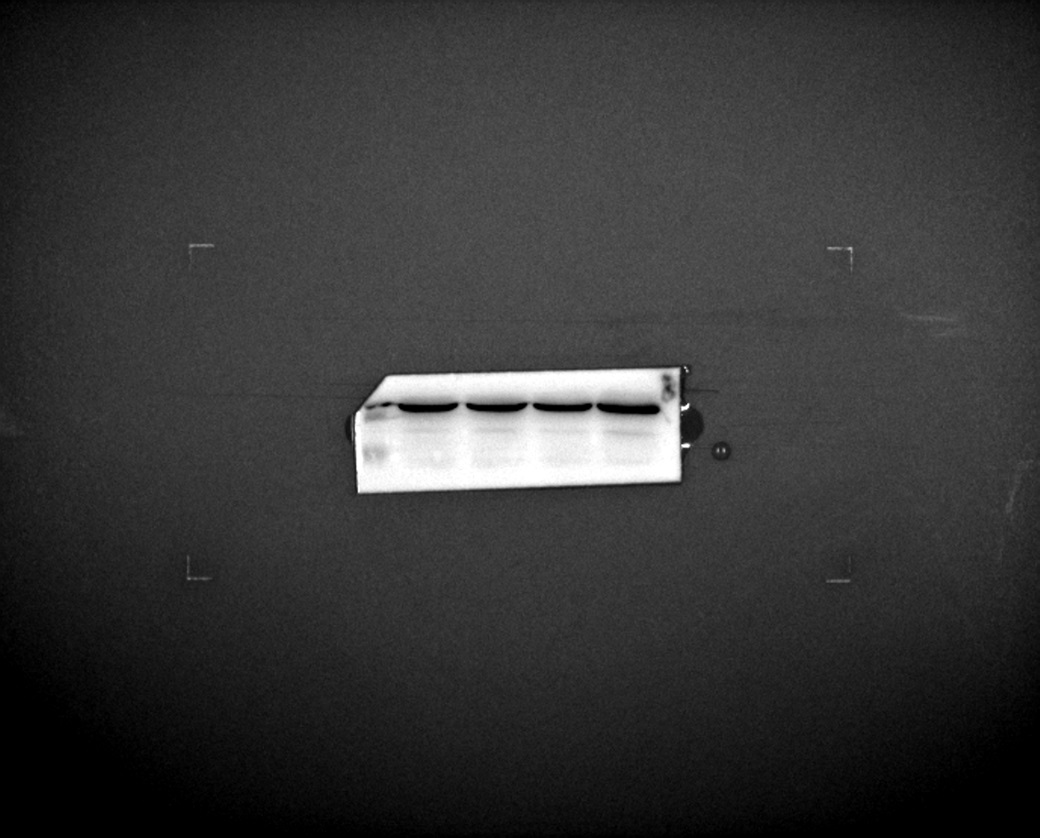
**

**（5）MAOA-3**

**
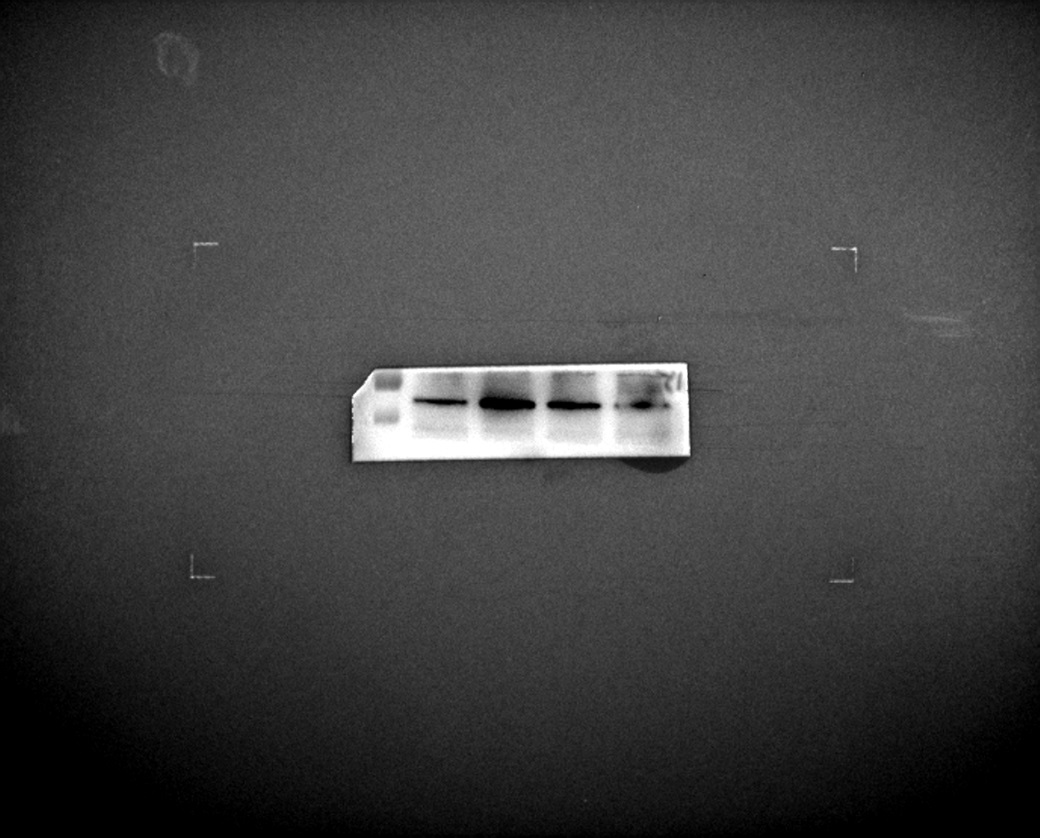
**

**（6）MAOA-β-actin-3**

**
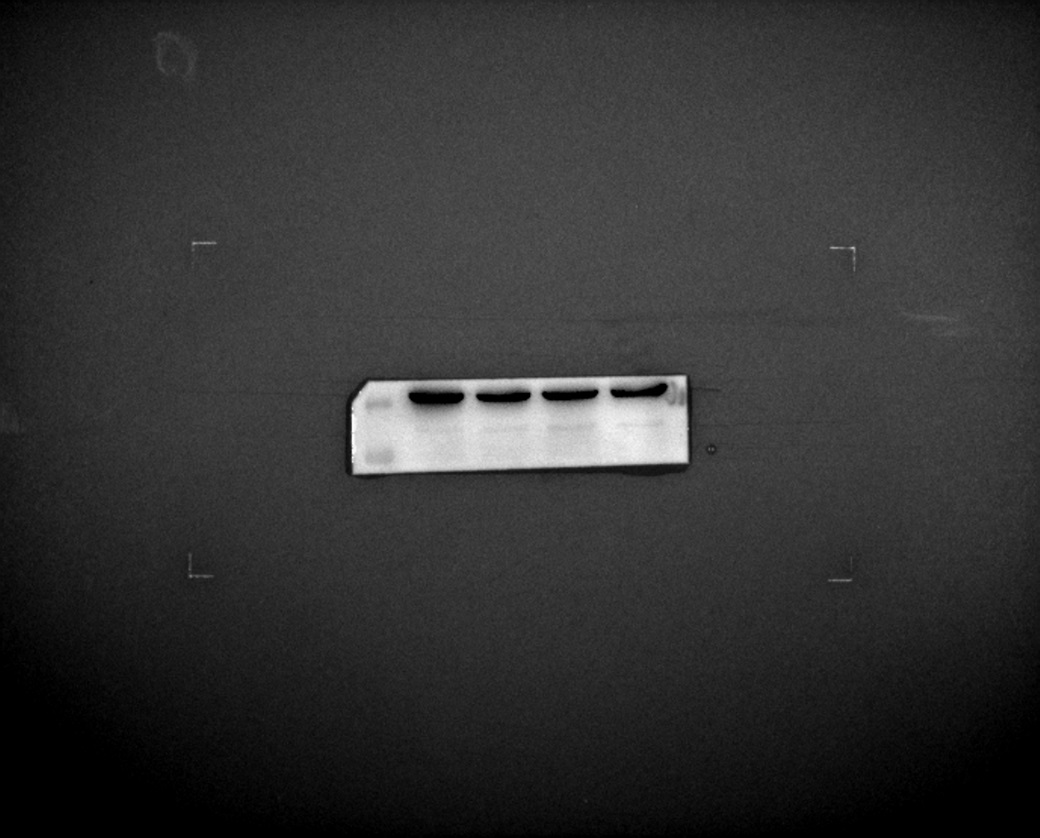
**

**二、PPARα**

**（1）PPARα-1**

**
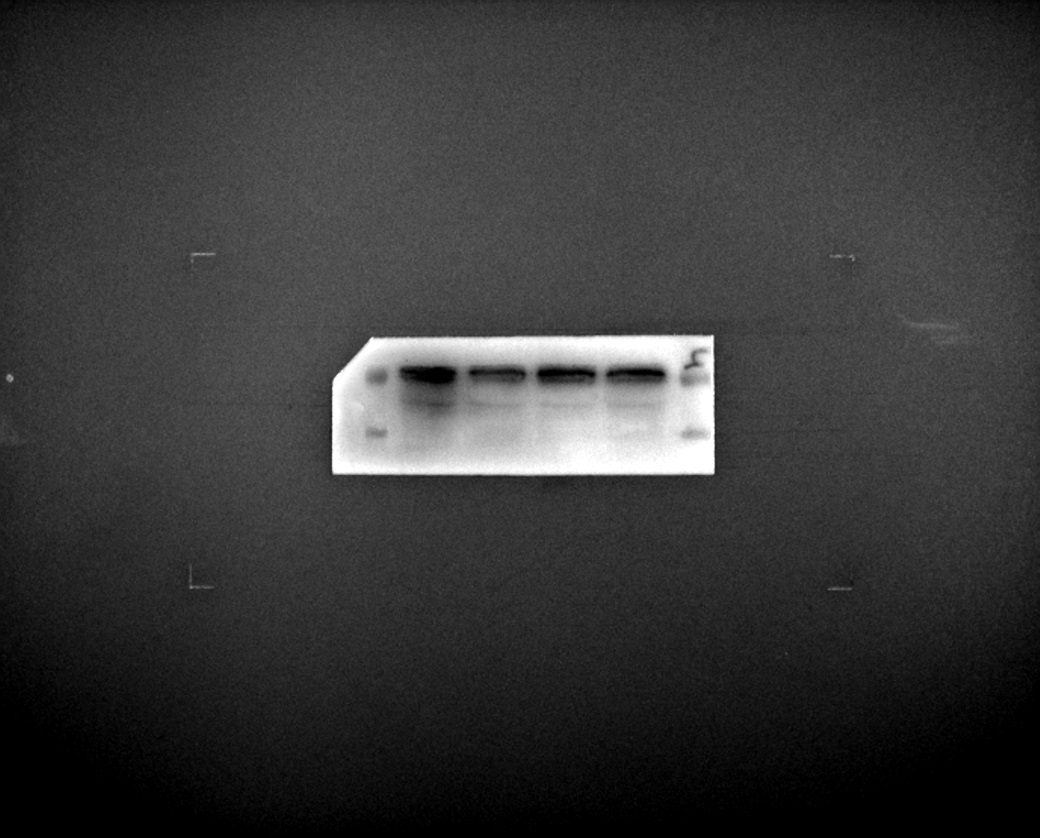
**

**（2）PPARα-β-actin-1**

**
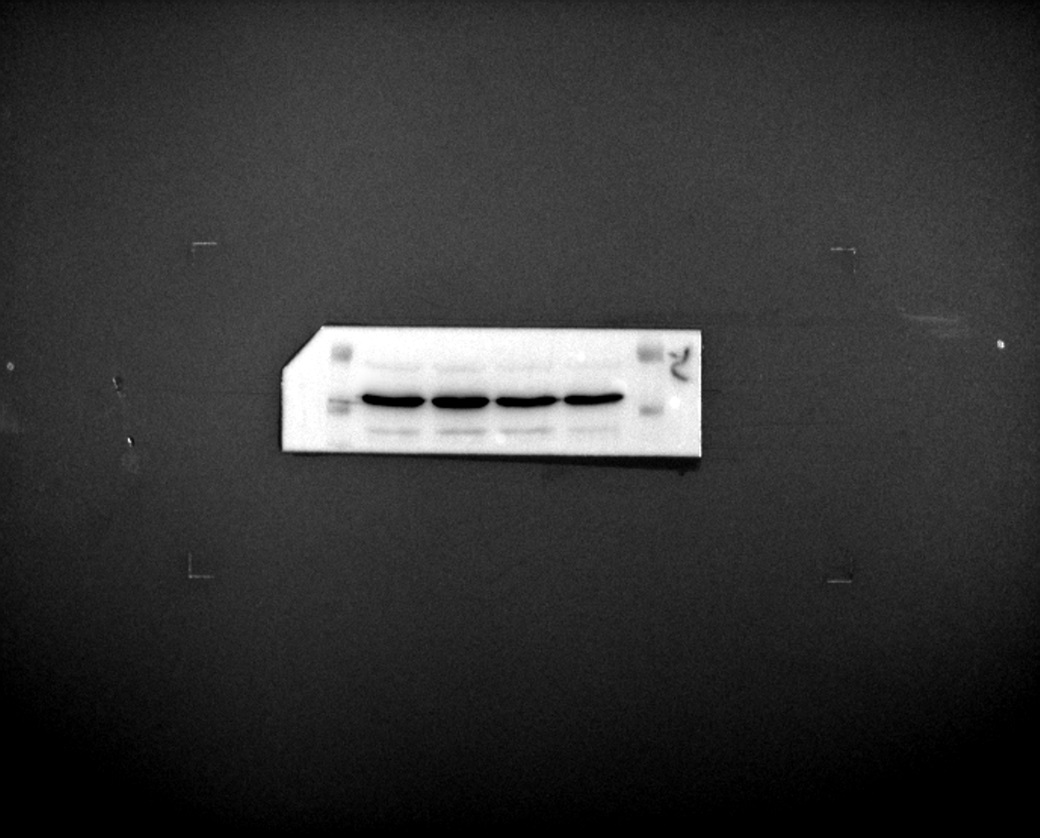
**

**（3）PPARα-2**

**
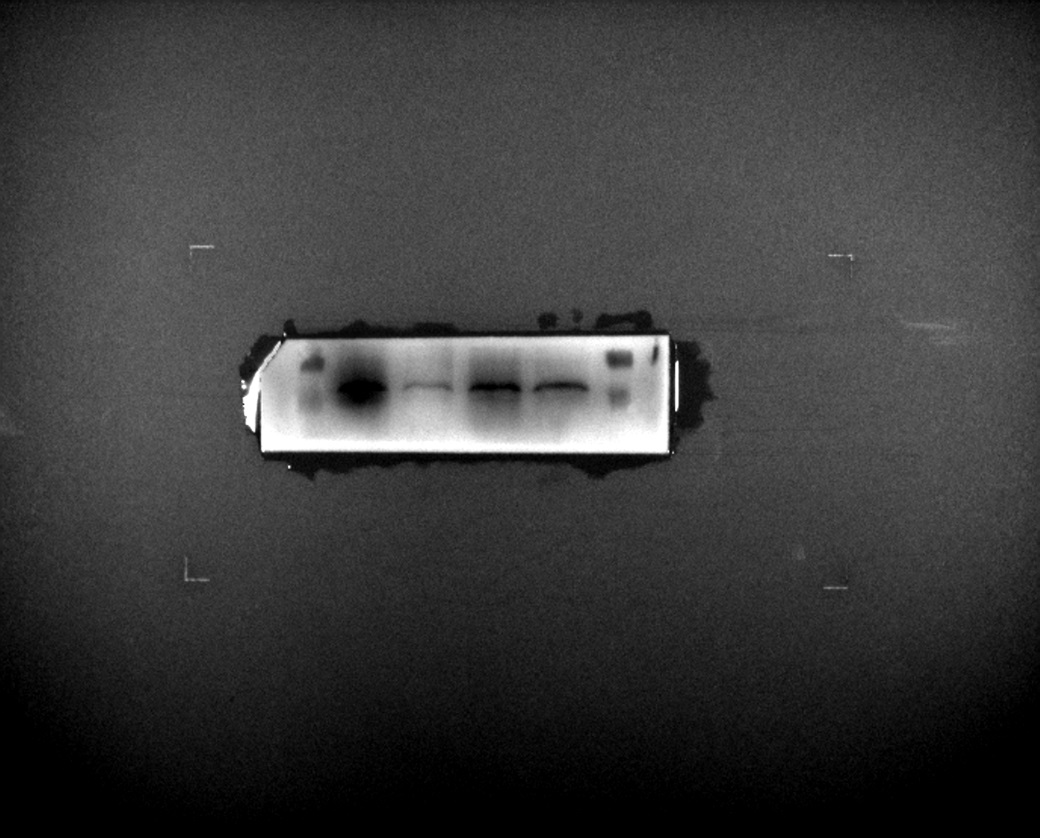
**

**（4）PPARα-β-actin-2**

**
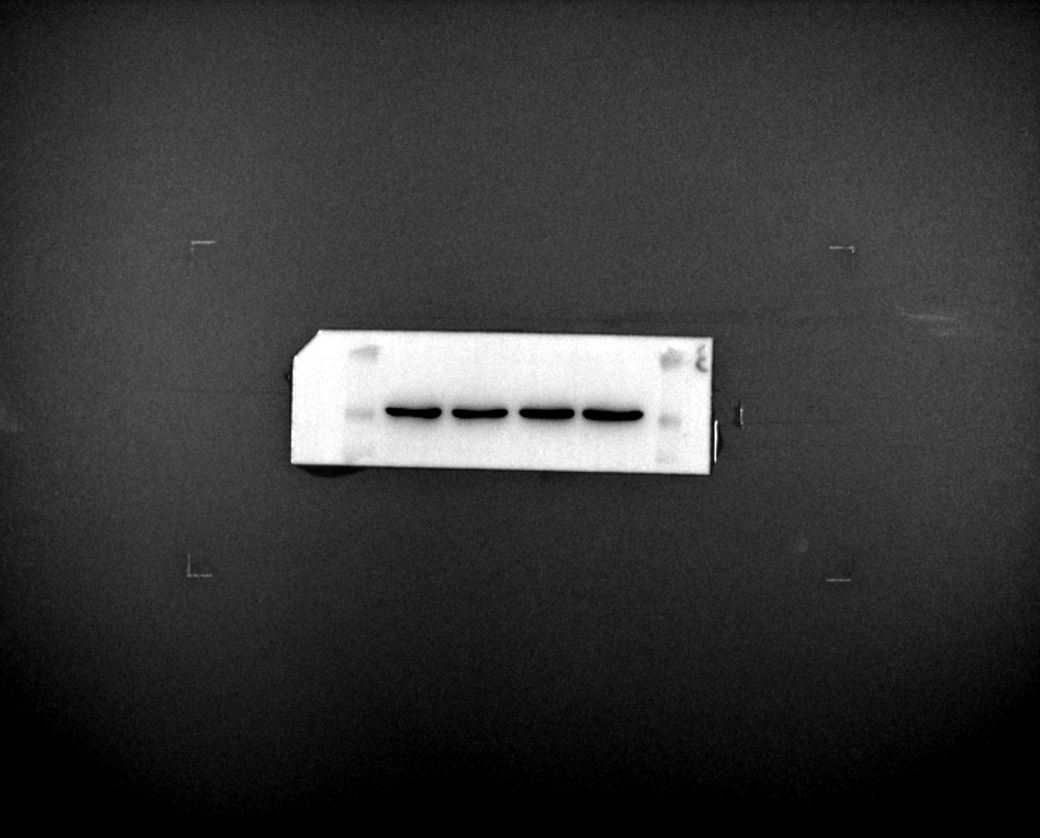
**

**（5）PPARα-3**

**
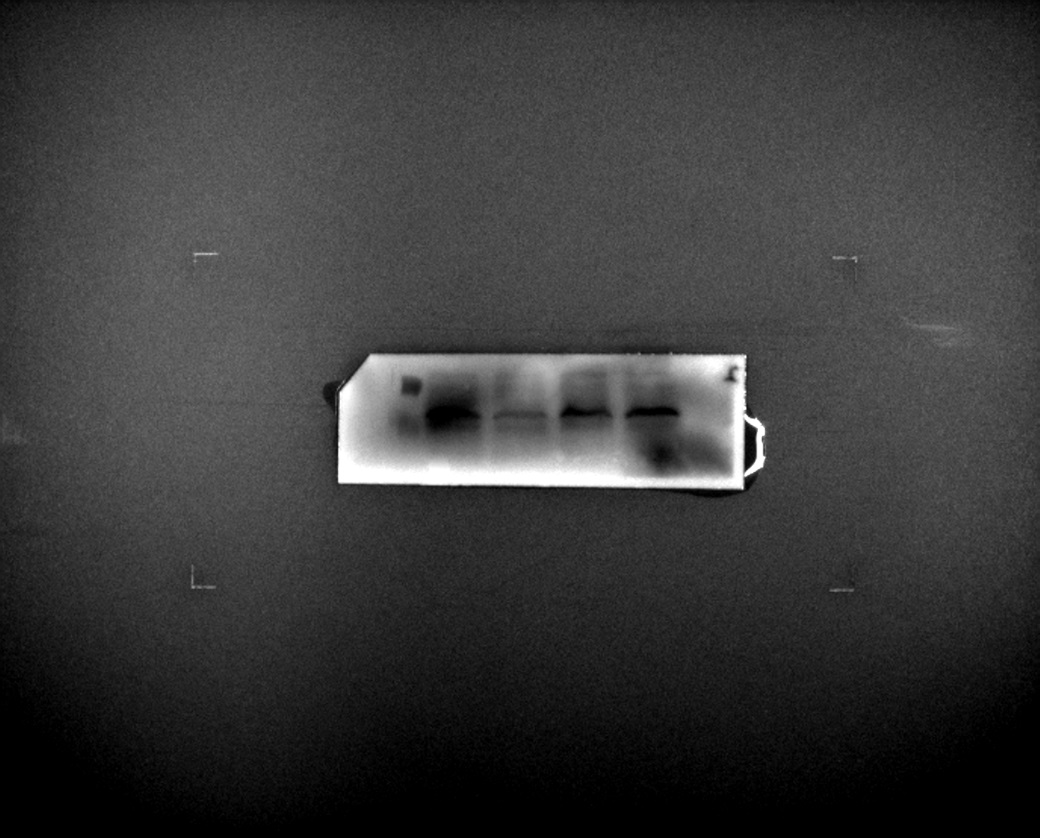
**

**（6）PPARα-β-actin-3**

**
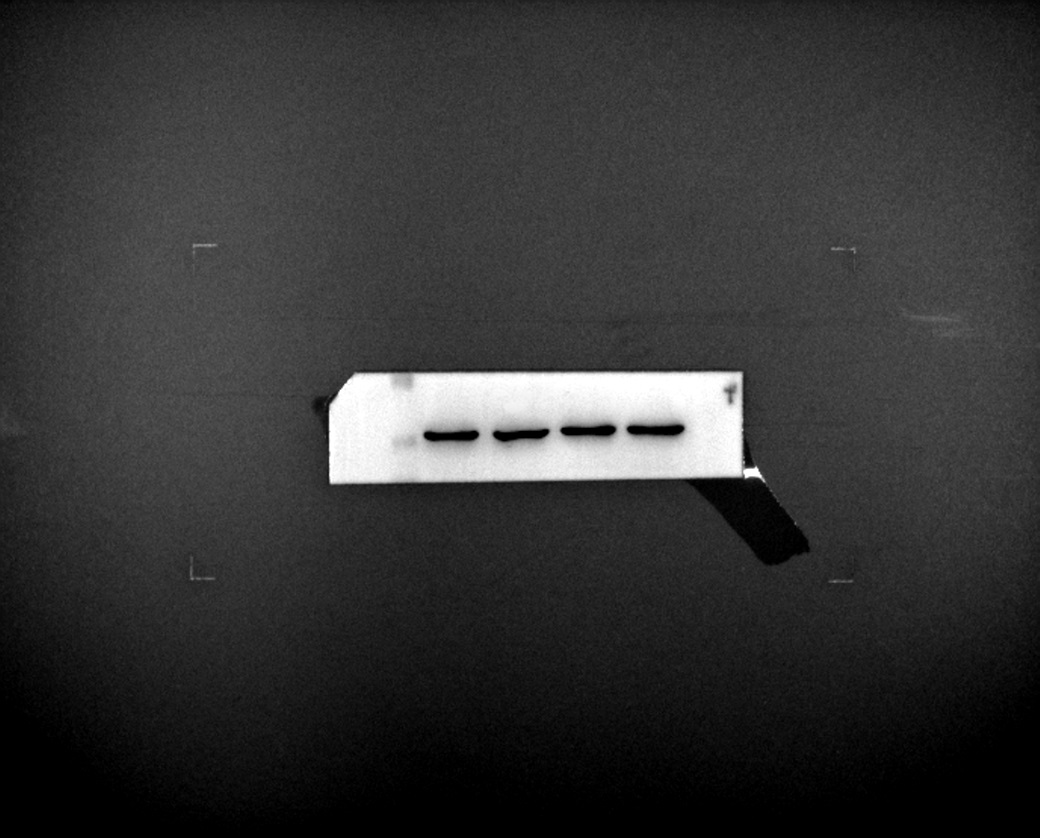
**
