# Supplementary material for: Systematic characterization of Puerariae Flos metabolites in vivo and assessment of its protective mechanisms against alcoholic liver injury in a rat model
Source: Front Pharmacol. 2022 Aug 30;13:915535. doi: 10.3389/fphar.2022.915535 (PMC9468746; doi:10.3389/fphar.2022.915535)
Supplement: Supplementary file 6 [file Table9.DOCX]

Dear Editor Heinrich

We appreciate your kind and instructive comments for our submitted manuscript (ID: 915535, Title: "Systematic characterization of the metabolites of Pueraria Flos in vivo and deciphering its protective mechanisms against alcoholic liver injury by a bioinformatics-integrated metabolic profile strategy and experimental validation"). The main corrections in the paper and the responses to your comments are appended below, which are also marked in red in the manuscript for better clarification.

1) The correct drug name should be 'Puerariae Flos' NOT 'Pueraria Flos'. Please check.

*In reply:*

Thank you for your suggestion. 'Pueraria Flos' has been replaced by 'Puerariae Flos', which has also been checked in the revised manuscript.

2) Replace 'network pharmacology' with 'network analysis'

*In reply:*

Thank you for your suggestion. 'network pharmacology' has been replaced by 'network analysis', which has also been checked in the revised manuscript.

3) Docking studies are not accepted unless followed by benchwork assessing affinity. A proposed mechanism of action is required. The compound you identified is a small molecule and these tend to show non-specific ‘docking’ effects. Further evidence is needed to ascertain the scientific plausibility of this claim. It is highly implausible that, for example, kaempferol, has any specific effects as you claim.

*In reply:*

Thank you for your question. As you suggested, benchwork assessing affinity using several technologies such as surface plasmon resonance (SPR) biosensor was the scientific strategy to evaluate and validate the docking study. While our research focus on the *in vivo* metabolites, the phase II metabolites including glucuronides, sulfates and/or bis-conjugates that account for the most part were difficult to isolate due to their exclusivity distribution in biological matrix rather than plant kingdom. Therefore, the absence of metabolite references restricted further affinity evaluation.

In our manuscript, network pharmacology was firstly employed to screen the relevant targets. As the importance of the screened targets by the method was sorted by the number of information included in the database. The absence of quantitative parameters such as binding energy could not reflect the structure-activity relationship and importance of targets. Thus, the docking analysis was performed to evaluate the relationship between the active components and potential targets. Finally, the increased or decreased expression of screened important targets PPARα and MAOA in rats with ALD model had verified the results of network pharmacology and molecular docking.

In the follow-up study, the problem you pointed out will be improved. We should try to purify the metabolites and employed the SPR assay to obtain the affinity and Michaelis constant of active compounds bond to key targets, which make the research more credible.

Besides, the limitation evaluation of this study was supplemented in the revised manuscript (P28, Line 21 ~ P29, Line 3) as follows, which had been marked in red for better clarification.

“There are still two shortcomings in this study. On the one hand, benchwork assessing affinity using several technologies such as surface plasmon resonance biosensor should be combined with docking analysis to evaluate and validate the importance of relative compounds and targets. As our research focus on the *in vivo* metabolites, the phase II metabolites including glucuronides, sulfates and/or bis-conjugates that account for the most part were difficult to isolate due to their exclusivity distribution in biological matrix rather than plant kingdom. Therefore, the absence of metabolite references restricted further affinity evaluation. On the other side, the single dose in the present study was chosen to continue and compare with our previous metabolism research, which determined the pharmacokinetic parameters of tectoridin and tectorigenin after oral administration at dosages of 200 and 130 mg/kg, respectively (Qu et al., 2012; Wang et al., 2013b). However, three dosages (low, middle and high) should be set to reflect the dose-effect relationship more scientifically in the pharmacological research. These deficiencies will be improved in our subsequent studies.”

4) Experimentally this is a single dose study, which in this case is fine, but this also needs to be spelled out as an important limitation.

*In reply:*

Thank you for your suggestion. The limitation was supplemented in the revised manuscript (P28, Line 21 ~ P29, Line 3) as follows, which had also been marked in red for better clarification.

“There are still two shortcomings in this study. On the one hand, benchwork assessing affinity using several technologies such as surface plasmon resonance biosensor should be combined with docking analysis to evaluate and validate the importance of relative compounds and targets. As our research focus on the *in vivo* metabolites, the phase II metabolites including glucuronides, sulfates and/or bis-conjugates that account for the most part were difficult to isolate due to their exclusivity distribution in biological matrix rather than plant kingdom. Therefore, the absence of metabolite references restricted further affinity evaluation. On the other side, the single dose in the present study was chosen to continue and compare with our previous metabolism research, which determined the pharmacokinetic parameters of tectoridin and tectorigenin after oral administration at dosages of 200 and 130 mg/kg, respectively (Qu et al., 2012; Wang et al., 2013b). However, three dosages (low, middle and high) should be set to reflect the dose-effect relationship more scientifically in the pharmacological research. These deficiencies will be improved in our subsequent studies.”

5) The MS still needs very careful editing for scientific clarity and correct grammar. Importantly, this also includes the abstract and title.

For example:

What is a 'medicinal and edible cognate'. A cognate is a word 'having the same linguistic derivation as another (e.g. English father, German Vater, Latin pater) (see Oxford Dict.) and this is clearly not correct here.

*In reply:*

Thank you for your advice. According to your suggestion, the title has been revised to “Systematic characterization of Puerariae Flos metabolites *in vivo* and assessment of its protective mechanisms against alcoholic liver injury in a rat model”. In the abstract, a 'medicinal and edible cognate' has been revised as 'a homology plant of medicine and food for alcoholism'.

Besides, the language has been modified sentence by sentence. And the names of metabolites in the whole text, Table 1 and Figure 2 was also standardize and unified.

6) What do you think about the following title: 'Pharmacokinetic characterization of Puerariae Flos metabolites and in vivo assessment of its protective mechanisms against alcoholic liver injury in a rat model.'

*In reply:*

Thank you for your advice. To the best of our knowledge, pharmacokinetic is a subject that mainly studies quantitatively the process (absorption, distribution, metabolism and excretion) and dynamic law of drugs in the organism. As the biosamples in our research were the combined ones without dynamic change process, so the ‘pharmacokinetic’ was not suitable. And the other revision could be accepted. The revised title would be employed as “Systematic characterization of Puerariae Flos metabolites *in vivo* and assessment of its protective mechanisms against alcoholic liver injury in a rat model”.

7) Several of the figures (e.g. # 3) are impossible to read and thus meaningless in scientific terms. It does not help to provide higher quality versions, but you must reconsider how you present the data.

*In reply:*

Thank you for your advice. The structure and description of Figure 3 has been recomposed and reorganized as follows, which had also been marked in red in the revised manuscript (P19, Line 11 ~ P20, Line 3) for better clarification.

“***3.3. Compound-target-pathway network construction***

In order to understand important effective components, the relative content of each metabolite was calculated by area normalization and expressed as the percentage of its peak area to the total peak areas in each kind of bio-samples (Table 1). 13 candidate components that detected in rat plasma and/or bile samples with relative content more than 3% were screened for the further network analysis. In all, 104 protein targets associated with the 13 constituents were retrieved from Swiss Target Prediction platform after eliminating the overlaps and a component-target network was constructed (Figure 3A). Their detail information is shown in Supplementary Table S2. Similarly, 5338 ALD-related targets obtained from OMIM, TTD, CTD, GAD, DisGeNET and GeneCards Database were collected after searching, integrating and de-duplicating steps (Supplementary Table S3).

**Figure 3**. Network construction and correlation analysis. (A) Distribution characteristic between 13 candidate absorbed components and their predicted targets; (B) Venn analysis of targets between 13 candidate components and ALD; (C) The PPI network of 47 merged targets according to the STRING database; (D) The interaction network of absorbed constituents, candidate targets and enriched pathways. A node stands for a constituent (left), a target (middle) or a pathway (right), the interactions of two nodes were represented by a line and the bigger size of a node refers to a higher degree.

To acquire the candidate targets of PF against ALD, above constituent-targets and disease-targets were intersected, and 88 intersection targets were obtained (Figure 3B). Then, protein-protein interaction (PPI) analysis aiming at more crucial targets was carried out based on the above 88 targets by using STRING database. Accordingly, 47 candidate targets with confidence scores greater than 0.9 were screened out (Figure 3C) and subjected to the KEGG pathway enrichment for elucidate the molecular mechanisms. As a result, 9 signaling pathways (excluded cancer pathways) were involved and enriched from 16 of 47 targets (Figure 3D), which could be sorted into four groups according to their biological functions: (1) glycolysis/gluconeogenesis related targets (LDHB, TPI1 and ALDH2); (2) amino acid metabolism related targets (ALDH2, GOT2, MAOA, MIF and TYR); (3) lipid regulation related targets (ALDH2, ACOX1, CHKB, PPARA and TNF); (4) inflammation and immune regulation related targets (TNF, HSP90AA1, HSP90B1, ALOX12, ALOX15 and PTGS1). Further, we mapped the 16 targets into components, and obtained 11 absorbed components (P6, P15, M3, M11, M15~M17, M19, M21, M36 and M55) (Figure 3D). ”

8) The conclusions only provide a summary and instead you need to include a critical assessment of what you have achieved AND what are the limitations. What sort of specific future research do you suggest?

*In reply:*

Thank you for your advice. We reconsidered our original conclusion and found it really failed to touch the quintessence of the whole research, so we reorganize the descriptions of conclusion as follows, which is also marked in red in the revised manuscript (P29, L4~ L16).

“**4.	Conclusions**

The present study has developed a sensitive and rapid method for the separation and identification of the absorbed constituents and metabolites of PF *in vivo* for the first time. Glucuronidation, sulfation, methylation, hydroxylation and reduction are the major metabolic reactions. Further constructed absorbed constituent-target-pathway-disease network and docking analysis revealed that phase II metabolites may play more important roles in the PF mediated protection against ALD. And the protective effects and predicted mechanism associated with decreased and elevated expression of MAO-A and PPAR-α in rat ALD models were also validated (Figure 7). However, the absence of metabolite references restricted the reliability of predicted conclusions about importance of key metabolites. In the future, we will try to purify the glucuronides, sulfates and/or bis-conjugated metabolites and employed the benchwork assay to obtain the affinity and Michaelis constant of active compounds bond to key targets, which make the research more credible.”

**Figure 7**. Overview of potential mechanisms underlying the protective effects of PF on alcohol-induced liver injury.

Thanks again.

With the regards

Dan Yuan, PhD & Prof. (yuandan_kampo@163.com)

Shenyang Pharmaceutical University
